# Structural Equation Modeling of Vocabulary Size and Depth Using Conventional and Bayesian Methods

**DOI:** 10.3389/fpsyg.2020.00618

**Published:** 2020-04-21

**Authors:** Rie Koizumi, Yo In’nami

**Affiliations:** ^1^School of Medicine, Juntendo University, Chiba, Japan; ^2^Faculty of Science and Engineering, Chuo University, Tokyo, Japan

**Keywords:** vocabulary size, vocabulary depth, factor structure, model testing, Bayesian structural equation modeling

## Abstract

In classifications of vocabulary knowledge, vocabulary size and depth have often been separately conceptualized (Schmitt, 2014). Although size and depth are known to be substantially correlated, it is not clear whether they are a single construct or two separate components of vocabulary knowledge (Yanagisawa and Webb, 2020). This issue has not been addressed extensively in the literature and can be better examined using structural equation modeling (SEM), with measurement error modeled separately from the construct of interest. The current study reports on conventional and Bayesian SEM approaches (e.g., Muthén and Asparouhov, 2012) to examine the factor structure of the size and depth of second language vocabulary knowledge of Japanese adult learners of English. A total of 255 participants took five vocabulary tests. One test was designed to measure vocabulary size in terms of the number of words known, while the remaining four were designed to measure vocabulary depth in terms of word association, polysemy, and collocation. All tests used a multiple-choice format. The size test was divided into three subtests according to word frequency. Results from conventional and Bayesian SEM show that a correlated two-factor model of size and depth with three and four indicators, respectively, fit better than a single-factor model of size and depth. In the two-factor model, vocabulary size and depth were strongly correlated (*r* = 0.945 for conventional SEM and 0.943 for Bayesian SEM with cross-loadings), but they were distinct. The implications of these findings are discussed.

## Introduction

The structure of language ability is a focus of concern for second language (L2) assessment researchers. Research on this issue dates back to Oller (1983), who reported on the unitary (i.e., single-factor) structure of a university placement test comprised of sections on grammar, composition, vocabulary, phonology, and dictation or cloze tasks. The findings of Oller’s study suggested that a single ability could be measured by a test consisting of multiple components of language ability. This finding was criticized by Bachman and Palmer (1983) and Farhady (1983), the whose results contradicted Oller and instead suggested that language ability consisted of multiple components. Research into the structure of language ability has continued, and numerous studies have made contributions to the issue (e.g., Shin, 2005; In’nami and Koizumi, 2012a; Sawaki and Sinharay, 2017; Yan et al., 2019).

Such investigations have for the most part focused on tests that assess skills (e.g., four skills in Sawaki and Sinharay, 2017; speaking in Sawaki, 2007; writing in Bae et al., 2016; listening and reading in Yamashita and Shiotsu, 2017). If the intended test constructs accord well with an observed factor structure, this constitutes one piece of evidence for the validity of interpretations based on test scores and evidence for an inference in the validity argument (e.g., Chapelle et al., 2008; Kane, 2013).

This line of research, which should also include vocabulary constructs (i.e., the vocabulary knowledge and ability that tests are intended to measure and what is actually measured; see Chapelle, 1998), has hitherto been limited. The necessity of investigating the quality of vocabulary tests and their constructs has been emphasized by Schmitt et al. (2020), who stated that L2 vocabulary fields need step-by-step test development and validation of vocabulary tests to allow for the meaningful interpretation and application of test scores.

While vocabulary knowledge has been conceptualized in various ways, vocabulary size and depth have often been separately conceptualized (Schmitt, 2014). Although the two have been shown to be substantially correlated, how size and depth should be conceptualized is not clear (Yanagisawa and Webb, 2020). Since they are strongly related to one another, should they be considered a single construct? Or should they be treated as two distinct constructs of vocabulary knowledge? These questions regarding the factor structure of size and depth can be better examined via structural equation models that take into account measurement error. Although structural equation modeling (SEM) has been used in language testing, models can be more flexibly tested using Bayesian estimation within the framework of SEM. The current study reports on the uses of conventional and Bayesian SEM to examine the factor structure of size and depth of L2 vocabulary knowledge of Japanese adult learners of English.

## Literature Review

### Defining Size and Depth

Many vocabulary researchers share the view that vocabulary knowledge can be classified into several components (e.g., Henriksen, 1999; Read, 2000; Meara, 2005; Daller et al., 2007; Milton, 2009; Schmitt, 2010; Nation and Webb, 2011; Webb and Nation, 2017; Nation, 2020). Of the several methods of classification, one in particular often used is the size and depth of vocabulary knowledge. It was proposed by Anderson and Freebody (1981) and is defined as follows: Size, or breadth, concerns a quantitative aspect related to knowledge of a word form and a primary meaning. This is also termed the form–meaning association. In contrast, depth involves a qualitative aspect associated with “how well a learner knows individual words or how well words are organized in the learner’s mental lexicon” (Staehr, 2009, p. 579).

Size has garnered much more attention as a research target than depth (Schmitt, 2014; Qian and Lin, 2020; Yanagisawa and Webb, 2020). In contrast, depth covers a wide range of lexical dimensions and is difficult to define. According to Webb (2013), “there is no definition of vocabulary depth that is widely agreed upon” (p. 1657). One of the leading researchers in depth studies, Read (2004) classified depth into three aspects: precision of meaning, comprehensive word knowledge, and network knowledge. Schmitt (2014), in relation to relationships between size and depth, organized depth into seven aspects: “receptive versus productive mastery, knowledge of multiple word knowledge components, knowledge of polysemous meaning senses, knowledge of derivative forms (word family members), knowledge of collocation, the ability to use lexical items fluently, and the degree and kind of lexical organization” (p. 922). Nation (2013, 2020) offered a comprehensive list of vocabulary knowledge by using three categories (i.e., Form, Meaning, and Use), each of which is further classified into three aspects: (a) Form: spoken, written, and word parts; (b) Meaning: form and meaning, concept and referents, and associations; and (c) Use: grammatical functions, collocations, and constraints on use (e.g., register, frequency). Each aspect has receptive and productive dimensions. Among them, “form and meaning, concept and referents, and collocation,” which Webb (2013) considers to be assessed by the Word Associates Format (WAF; Read, 1993, 1998), seem to be the aspects studied most.

While both size and depth are important for language use, size has been considered the primary aspect of vocabulary knowledge because of its importance in the form–meaning link for vocabulary use (e.g., Laufer et al., 2004; Webb, 2005; Schmitt, 2010). Given the centrality of size, indications of form–meaning knowledge are often interpreted as having the ability to use words in reading, listening, writing, and speaking, and even as having vocabulary depth such as derivatives and collocations. However, Kremmel and Schmitt (2016) argue that these interpretations are not justified based on their research.

### Measuring Size and Depth

Size and depth have been measured using various formats. Size has been typically measured by means of a recognition (e.g., multiple-choice or matching) or recall (e.g., translation) format, in which the L2 target form or its meaning is presented and test takers select or supply the meaning or L2 form (e.g., Laufer et al., 2004). There exist many vocabulary size tests, such as the Vocabulary Levels Test (Nation, 1983; Schmitt et al., 2001) and the Vocabulary Size Test (Nation and Beglar, 2007). The following shows a sample item from the Vocabulary Size Test (Nation and Beglar, 2007, JALT2007, p. 2), in which test takers are asked to select the most appropriate meaning of the written target word out of four written choices.

**poor: We are poor.** (^∗^answer)

a. have no money^∗^
b. feel happy
c. are very interested
d. do not like to work hard

While the form–meaning association appears relatively simple to define and assess, research has shown that it is not: Size test scores are affected not only by the intended test construct but also by various factors such as differences in item formats and test takers’ test-taking strategies (Gyllstad et al., 2015; Kremmel and Schmitt, 2016; McLean et al., 2020).

Still, measuring size is less complicated than measuring depth. Depth is a multifaceted construct, ranging from various aspects of vocabulary to lexical organization, resulting in varied test formats. Yanagisawa and Webb (2020) grouped various approaches to measuring depth into three categories: a developmental approach, a lexical network approach, and a components approach. The developmental approach considers depth as something expanding from zero to full knowledge and attempts to test on what stage learners are located. An example can be found in the Vocabulary Knowledge Scale in Wesche and Paribakht (1993, p. 30), which uses self-assessment and some production items. Test takers are asked to indicate their degree of knowledge of each target word using the following scale.

I. I don’t remember having seen this word before.
II. I have seen this word before, but I don’t know what it means.
III. I have seen this word before, and I *think* it means.____ (synonym or translation)
IV. I *know* this word. It means.____ (synonym or translation)
V. I can use this word in a sentence:____________. (If you do this section, please also do Section IV.)

Scores vary according to the quality of written responses. For example, if the synonym or translation provided in III–IV by test takers is wrong, those who choose III gain a score of 2. Yanagisawa and Webb (2020) summarize validity issues related to the Vocabulary Knowledge Scale. These issues include the lack of empirical basis for the developmental scale structure and difficulty in interpreting total scores because the test assesses multiple aspects of vocabulary knowledge in different stages.

In the lexical network approach, depth is conceived as a lexical network in which words are associated in learners’ mental lexicon, and indications of knowledge of word association are elicited in tests taking this approach. The WAF (Read, 1993, 1998) uses this approach and is possibly the most frequently used depth measure (Yanagisawa and Webb, 2020). In the following sample item from Read (1998, p. 46), test takers are asked to select four words related to the stimulus word out of eight options. In the box on the left, words that may have paradigmatic associations with the cue word (synonym or one element of the meaning) are presented, whereas the box on the right contains words that may have syntagmatic associations with the cue word (collocations). There are possibly one to three answers out of four in the left box and one to three answers in the right box, and four answers in total.

**sudden**

beautiful quick^∗^ surprising^∗^ thirsty | change^∗^ doctor noise^∗^ school

While the WAF is relatively easy to administer and score, there are limitations: For example, this format taps limited aspects of the lexical network; this format allows test takers to use guessing strategies; studies using the WAF have modified test formats and scoring methods according to their research orientations, so the scores are not always comparable across studies (Yanagisawa and Webb, 2020); it is also rather difficult to interpret what its total scores mean because multiple aspects of vocabulary depth are combined (Webb, 2013).

The components approach handles different aspects of depth separately. Webb (2013) recommended this approach, stating that creating tests assessing each aspect separately would bring the field forward for more precise depth assessment and research. Using this principle, multiple measures have been developed. For example, Webb (2005) developed 10 tests that focus on five aspects (i.e., written form, form and meaning, association, collocation, and grammatical functions), each of which was assessed with receptive or productive (i.e., recognition or recall) formats. Tests focusing on written form assessed size, whereas those focusing on the other four aspects assessed depth. Nguyen and Webb (2017) developed a collocation test in a multiple-choice format in which test takers were required to choose “the word that co-occurred most frequently with the node word from four options” (p. 306). An example is shown below (p. 309).

**advantage** a. get b. give c. have d. take^∗^

Among the three approaches to measuring depth (i.e., the developmental, lexical network, and components approaches), Yanagisawa and Webb (2020) recommended the components approach most because of its transparency in what the test scores indicate. They suggested investigating a wider range of depth aspects by using separate tests. The current study responds to this call for research and develops tests separately focusing on three depth aspects: association, polysemy, and collocation.

### Correlations Between Size and Depth

Numerous researchers have examined the relationship between size and depth in L2 vocabulary studies (e.g., Nurweni and Read, 1999; Mochizuki and Aizawa, 2000; Vermeer, 2001; Noro, 2002; Qian, 2002; Akase, 2005; Shimamoto, 2005; Ishii and Schmitt, 2009; Koizumi and In’nami, 2013; Kremmel and Schmitt, 2016; see Schmitt, 2014, for a comprehensive summary). They have been interested in exploring the degree to which size and depth are related and how constructs of size and depth can be conceptualized in L2 vocabulary assessment. In his seminal article on a critical review of studies on vocabulary size and depth, Schmitt (2014) posed the following questions: “Do size and depth behave as separate constructs,” “or are they essentially the same construct?” (p. 941). These questions underlie the research conducted and discussions held thus far. For example, Akbarian (2010) reported a strong simple (zero-order) correlation (*r* = 0.864) between vocabulary size and depth among 112 Iranian learners of English. Size was measured using the Vocabulary Levels Test (Schmitt et al., 2001), whereas depth was measured using the WAF (Read, 1993). Strong correlations were also found in Vermeer (2001). He examined 25 L2 Dutch kindergarteners who took two size tests in which words were presented orally. In one test, they selected the picture option that showed the meaning of the word they heard; in the other, they described the meaning of the word presented. In the depth test, they were asked to express what they knew about the target word by answering the following questions: “What is a …?” “What does a … usually look like?” “What can you do with a …?” “What do you feel when you touch a …?” and “Can you tell us some more about a …?” (p. 224). Their responses to the depth test were evaluated in terms of the quality of the word association network. It was found that the size test scores strongly correlated with the depth test scores (*r* = 0.72–0.76), which led him to state that “there is no conceptual distinction between the two” (p. 231). Schmitt (2014) attributed these high correlations to overlapping constructs. He argued that “the depth test only tapped into deeper semantic knowledge of a single meaning sense, so all tests (both size and depth) were essentially various types of meaning tests” (p. 921). He added that if he had used the measures that assess broad aspects of depth, the correlations would not have been so strong.

This hypothesis has been supported by previous studies such as Schmitt and Meara (1997), which examined the relationships between size and depth (i.e., both receptive and productive aspects of word association and suffix knowledge) among 88 Japanese learners of English. Size was assessed by the Vocabulary Levels Test (Nation, 1983). Receptive word association and suffix knowledge were assessed by requiring test takers to select the correct suffixes and words associated with a target word. Productive word association and suffix knowledge were assessed by requiring test takers to write every suffix that they thought could be added to the stimulus word as well as three word associations prompted by the stimulus. The simple correlations between size and depth aspects ranged from low to moderate (*r* = 0.27–0.62).

Findings from previous studies suggest that size and depth are correlated but that the strength of correlations varies from weak to strong across studies. Schmitt (2014) has attributed this variation mainly to different types of depth assessed and instruments used and to different L2 proficiency levels of test takers. He also pointed out that many depth tests may have problems related to reliability and validity. Since correlation coefficients are lowered in tests with low reliability, measurement error may partly explain the differing strengths of the relationships between size and depth across studies. One way to more accurately estimate correlation coefficients while addressing measurement error is to use SEM. SEM has been used to examine the factor structure of language ability by testing the fit of models to data. Ability and measurement error are modeled separately so that the relationships between abilities can be more precisely examined while separately estimating the impact of measurement error (see In’nami and Koizumi, 2011; Winke, 2014; Ockey and Choi, 2015, for SEM in an L2 assessment field).

In vocabulary studies, the factor structure of size and depth can be modeled using SEM in two ways. First, in a single-factor model, both size and depth measures (i.e., observed variables) are hypothesized to reflect one vocabulary factor (size and depth combined). If this model is the most likely, the distinction of size and depth is not very important, as size and depth assess the same vocabulary knowledge. Second, in a correlated factor model, size and depth factors are hypothesized to be correlated with one another. Even when they are correlated very highly, they should be treated separately, as doing so better explains the data.

A few previous studies examined a factor structure of the L2 vocabulary size and depth of L2 learners by modeling both size and depth as latent factors and comparing fit statistics across multiple models: Tannenbaum (2008) targeted first language (L1) users, and Kieffer and Lesaux (2012) targeted L1 and L2 users and analyzed a combined sample. To our knowledge, Vafaee (2016) is the only study that focuses on L2 learners’ vocabulary factor structure. The authors are aware that several studies used SEM but did not model size or depth as a separate latent factor (Tseng and Schmitt, 2008; Zhang, 2012; Koizumi and In’nami, 2013), or one study (Tseng, 2011, as cited in Schmitt, 2014, p. 930–931) did not provide sufficient information for review.

Vafaee (2016) examined the relationship between size and depth of 263 lower-intermediate to advanced Persian learners of English. In the size test, test takers listened to a word and non-defining sentence once and selected from four choices of L2 meanings provided on the answer sheet (i.e., an aural version of the Vocabulary Size Test; Nation and Beglar, 2007). The test was divided into four sections according to the frequency of target words, and these four sections were used as indicators of vocabulary size (α = 0.67–0.84). In addition, an aural test of depth was created by adapting the WAF (Read, 1993, 1998). Test takers listened to the target word and options and were required to choose a synonym or collocation in relation to the target word. Results of synonym and collocation were separately scored, with synonym and collocation forming two indicators of depth (α = 0.92–0.93). There were moderate simple correlations between size and depth indicators (*r* = 0.64–0.77). A single-factor model with six indicators of size and depth was compared to another model (size and depth were separately modeled and correlated). The latter model (a correlated two-factor model) fit the data better than the single-factor model, with size and depth highly correlated (*r* = 0.94). However, the results of Vafaee (2016) may have been affected by (a) measures used to assess size and depth, (b) aspects assessed by depth tests, or (c) other features, such as participants’ L1 and L2, or L2 proficiency levels. Regarding (a), Vafaee (2016) used aural versions of the Vocabulary Size Test and the WAF. Regarding (b), the research focused on synonym and collocation, as measured by the WAF. Although an aural version of the WAF was developed for the research, issues related to WAF test interpretation and use mentioned in the *Literature Review* apply to this research as well. Regarding (c), the participants were Persian learners of English at lower-intermediate to advanced levels. In order to know to what extent the findings of Vafaee can be generalized beyond contexts, further research is needed to examine a factor structure of size and depth with different types of measures addressing different aspects of the vocabulary knowledge of various target participants. Thus, this study examines a factor structure of size and depth, targeting beginner to intermediate Japanese learners of English, using diverse measures of depth.

## Current Study

To examine the relationship between the size and depth of L2 vocabulary knowledge, the following research question is investigated in the context of L1 Japanese adult learners who studied English as a foreign language at beginner to intermediate levels.

Research question: Which factor structure of the size and depth of vocabulary knowledge explains the data better, a single-factor or a correlated two-factor model?

The current study expands on the findings of previous studies in five ways: First, we include three aspects of depth for analysis: word association, polysemy, and collocation. As described in the *Literature Review*, many previous studies, including Vafaee (2016), have used the WAF, which basically targets synonym and collocation; we increase the number of the aspects of vocabulary depth measured from two to three by employing more tests. We intentionally select word association, polysemy, and collocation, which are more closely related to a form–meaning link than are other depth aspects such as word parts, to rigorously examine the separability of size and depth constructs. Second, we use four separate depth tests by taking the components approach. Third, unlike studies that used simple correlations or regressions to investigate the relationship between size and depth (e.g., Vermeer, 2001; Akbarian, 2010), we use SEM to empirically identify the structure that best fits the data. The use of SEM should more clearly elucidate the relationship in question, with the measurement error of the instruments examined separately. Fourth, we explicitly compare a single-factor model with a correlated two-factor model using SEM. The identification of a best-fitting model of size and depth in comparison to competing models would have strong implications for vocabulary theory and practice. Fifth, we use both conventional and Bayesian SEM. In conventional SEM (and particularly in confirmatory factor analysis), the relationships between observed variables and factors are modeled by specifying paths between the two. Specifying no path indicates that no such relationship is hypothesized. According to Muthén and Asparouhov (2012), this is a very strong assumption and may not reflect researchers’ theories or hypotheses since it is highly unlikely that no relationship exists between observed variables and factors. They have stated that it would be more sensible to model near-zero relationships with some variability between these observed variables and factors. Yet, conventional SEM does not allow researchers to specify such models. This is possible in Bayesian SEM, where degrees of a relationship can be specified using prior information (i.e., priors) based on theory and previous studies. This allows for more flexible testing of models by enabling researchers to specify major and minor loadings, namely those expressed as near-zero cross-loadings and correlations between residuals (i.e., measurement error).

## Method

### Participants

In 2012, a total of 255 adult learners (18 or older) took vocabulary tests as part of their L2 English courses or as volunteers. Originally, 257 students took the tests, but 2 were found not to have taken the test seriously, so these 2 students were not included. Of these 255 test takers, 239 were undergraduates at nine Japanese universities; 9 were graduate students at four Japanese universities; and 7 were professionals who used English frequently. The undergraduate and graduate participants attended national or private universities and majored in various subjects. Other information such as gender and age was not available, but it is reasonable to assume that most participants were 18–22 years old and studied English as a foreign language for at least 6 years at the secondary school level. This is because most undergraduates in Japan are in this age range and have similar English-learning experience.

They took five vocabulary tests (see *Instruments and Procedures* below) and provided scores obtained in 2012 or earlier for the TOEIC (Test of English for International Communication^®^) Listening and Reading Test. The distribution of participants’ TOEIC scores (*M* = 514.84; *SD* = 181.17; Min = 205; Max = 985) resembled the distribution of all Japanese test takers for the TOEIC test (*M* = 520; *SD* = 180; reported in Educational Testing Service, 2019). Most participants were estimated to possess an A2 level proficiency of the Common European Framework of Reference (CEFR; Listening = 62.75%; Reading = 50.20%), based on their TOEIC Listening and Reading Test scores and a conversion table (Tannenbaum and Wylie, 2013).

### Instruments and Procedures

The intent of the study’s five vocabulary tests was to measure vocabulary size (one test) and vocabulary depth (four tests). We used a multiple-choice format with four or five options (see Table 1 for examples and Appendix A in Supplementary Material for all the test items). All the tests employed a discrete, selective, context-independent format (Read, 2000). Words used in the size and depth tests were different across tests.

**TABLE 1:** Examples of the five vocabulary tests in order of administration.

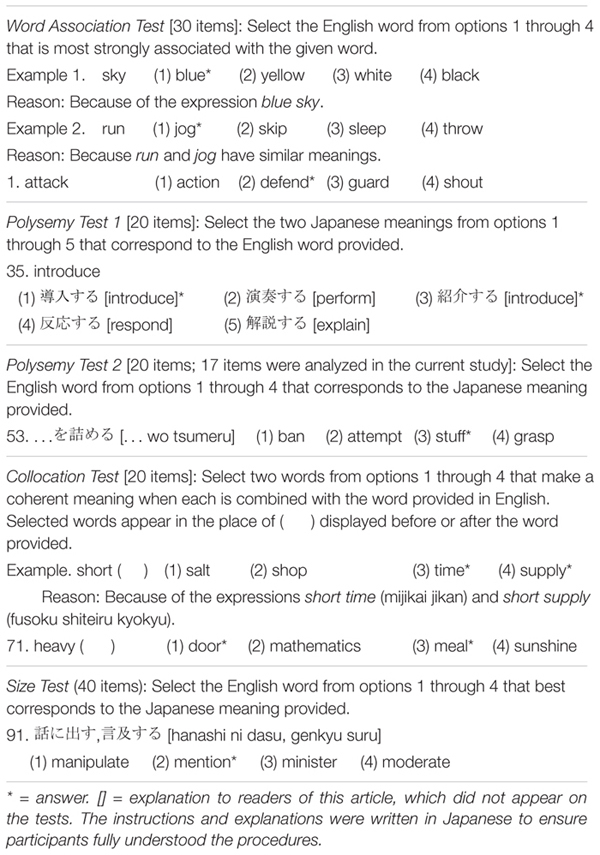

The tests were developed for research, using lemma as a basis of counting units (see Vilkaitė-Lozdienė and Schmitt, 2020, for its appropriateness). They were constructed using the JACET8000 vocabulary list, a word list specifically adapted to Japanese learners of English [JACET (Japan Association of College English Teachers) Basic Word Revision Committee, JACET Basic Word Revision Committee, 2003]. This list was compiled using the British National Corpus (BNC) and subcorpora based on material that Japanese learners of English are likely to encounter, such as in textbooks for secondary schools. We considered using the word list matching the target learners’ learning context to be appropriate for measuring their vocabulary (Nation and Sorell, 2016). Readers can refer to Appendices B, C in Supplementary Material for information on word frequency. The JACET8000 vocabulary list was later updated (JACET Basic Word Revision Committee, 2016; see the older and latest version lists^1^). All the tests were piloted and revised before the validity of interpretations based on the scores of each test was examined and reported in Mochizuki et al. (2014).

The JACET8000 Vocabulary Size Test was intended to assess lexical knowledge of L2 written forms and the primary meanings (the first definition that appears in dictionaries) of up to 8,000 lemma. Test takers were required to select an L2 form that corresponded to a meaning provided in L1 Japanese. There were 40 items in total, with 5 items for each 1,000-lemma level. The 40 items were divided, according to word frequency, into three subtests of 15 (levels 1,000–3,000), 15 (4,000–6,000), and 10 items (7,000–8,000).

The second through the fifth vocabulary tests assessed three aspects of depth of vocabulary knowledge: word association, polysemy (two formats), and collocation. Stimulus words presented in each test were selected from the 1,000- to 3,000-lemma levels (Polysemy Test 2) or from the 1,000- to 2,000-lemma levels (the other three tests). All correct options but one (Word Association Test, No. 12) were within 3,000-lemma levels (see Appendix B in Supplementary Material).

In the 30-item Word Association Test, test takers were required to choose which L2 word was associated the most strongly with the L2 word provided. To construct this test, Mochizuki et al. (2014) asked Japanese learners of English with low to high proficiency to write three to five English words related to stimulus words (e.g., *sky*). They then selected (a) a word association as an answer that distinguished low- and high-level learners and (b) distractor word associations that did not distinguish between the two levels.

There were two polysemy tests. The first (Polysemy Test 1, 20 items) asked test takers to select two frequent meanings of an L2 polysemous word (including homographs). They were selected from the following lists of polysemous words (Gorfein et al., 1982; Twilley et al., 1994; Seto, 2007).

The other polysemy test (Polysemy Test 2) required test takers to choose an English word with the same meaning in Japanese. The stimulus words were selected from words that had at least three meanings displayed in the *Collins COBUILD Advanced Learner’s English Dictionary*. The definition that appeared third in the dictionary was selected. Of the 20 items originally on the test, only 17 of the items were used for analysis because the remaining 3 items were found to assess knowledge of the first definition, which overlapped the concept of vocabulary size. It should be noted that the Size Test and Polysemy Tests 1 and 2 all assessed relationships between L2 form and L1 meaning but differed in their constructs in that the Size Test assessed primary meanings with higher frequency, Polysemy Test 1 assessed two frequent meanings, and Polysemy Test 2 assessed a less frequent meaning.

Finally, in the 20-item Collocation Test, test takers were required to select two L2 words that co-occurred with the L2 word provided. The collocation was either of an adjective + noun type or of a noun + noun type; these were selected from the *Longman Dictionary of Contemporary English* (5th ed.) and *Oxford Collocations Dictionary for Students of English*. Distractors were selected from words that least collocated with the stimulus word, and this assumption was confirmed by asking two experienced Japanese teachers of English and an English native speaker. We also examined the items by mutual information (MI) scores using the Corpus of Contemporary American English (COCA) and BNC, accessed through English-Corpora.org^2^, and found no major problems (see Appendix C in Supplementary Material for details). As collocation is a part of word association, the Collocation Test and Word Association Test partially overlap the constructs. However, we intended to assess wider areas of depth of vocabulary knowledge instead of avoiding the overlaps.

For the Polysemy 1 and Collocation Tests, one point was awarded when two correct options were selected, whereas for the Size, Word Association, and Polysemy 2 Tests, one point was awarded when the correct option was selected.

### Analysis

**FIGURE 1:**
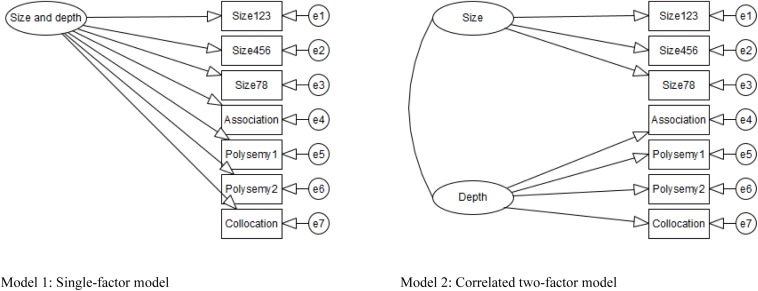
Models of vocabulary size and depth using conventional structural equation modeling (SEM). Size123 = size with 1,000–3,000 levels; Size456 = size with 4,000–6,000 levels; Size78 = size with 7,000 and 8,000 levels. All figures in this article were created using yEd Graph Editor (Version 3.19.1.1; yWorks GmbH, 2000–2019).

**FIGURE 2:**
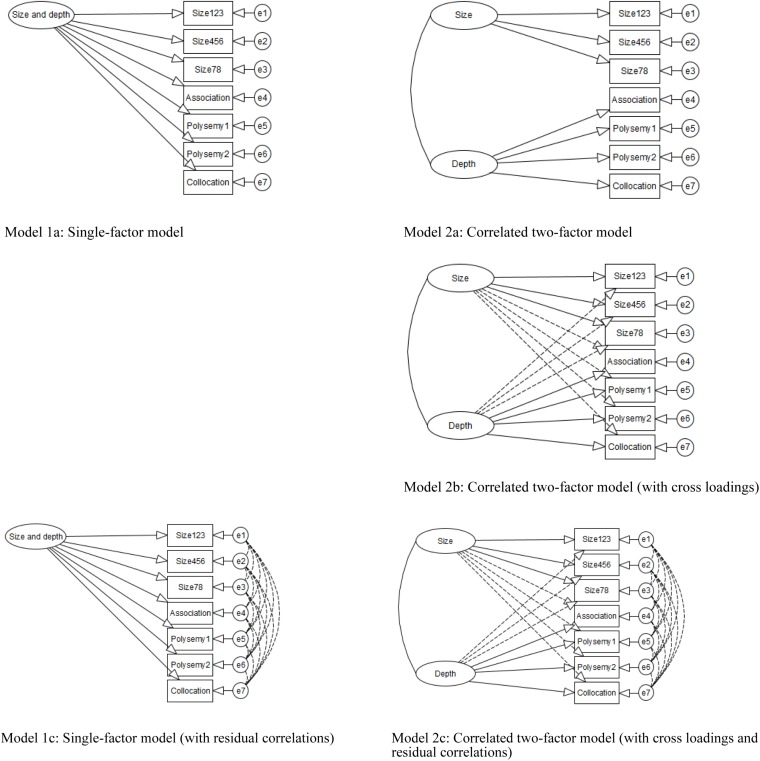
Models of vocabulary size and depth using Bayesian SEM. Undotted line = major loading; dotted line = minor loading. All residuals are correlated in Models 1c and 2c.

The structure of the size and depth of vocabulary knowledge was examined by testing two variants of models that hypothesized the relationships among variables as single-factor or correlated two-factor models. These models are presented in Figures 1, 2. In each figure, the rectangles represent observed variables, the ovals represent latent factors, and the circles represent measurement errors or residuals. Models 1 and 2 were built based on the structures of vocabulary knowledge discussed in the literature (e.g., Vafaee, 2016). Model 1 had three indicators of size and four indicators of depth. Both size and depth were hypothesized as a single factor of vocabulary knowledge. In Model 2, the same indicators were used to hypothesize correlated but separate factors of size and depth (see details of the models below).

The observed variables in this study were composite scores aggregated using item-level dichotomous data. The unidimensionality of each observed variable was examined and confirmed before the aggregation (e.g., Little et al., 2002, 2013; Meade and Kroustalils, 2006).

After a preliminary analysis of score distribution and reliability, conventional and Bayesian SEM was conducted using Mplus (Version 8.3; Muthén and Muthén, 1998–2019; see Appendices D–H in Supplementary Material for Mplus codes used). There were no missing values. For scale identification, one loading from a factor was fixed to 1.00. Observed variables were standardized to ease interpretation of priors (Muthén and Asparouhov, 2012). For conventional SEM, the data were univariately normally distributed, as judged by the skewness and kurtosis values of |3.30| (the *z* score at *p* < 0.01; e.g., Tabachnick and Fidell, 2014) and histograms. The data were multivariately non-normal according to Mardia’s multivariate normality test available in an R package, MVN (Korkmaz et al., 2019). To account for such multivariate non-normality, a maximum likelihood estimation with a robust standard errors method was employed for estimation. Models were judged using fit indices: a comparative fit index (CFI) of 0.90 or above (Arbuckle and Wothke, 1995), a root mean square error of approximation (RMSEA) of 0.08 or below, and a standardized root mean square residual (SRMR) of 0.08 or below (Hu and Bentler, 1999). The Akaike information criterion (AIC) and chi-square difference tests were used to compare models (see Mplus, 2019). With statistical non-significance, a more parsimonious model with fewer parameters to estimate (usually, a model with a greater number of degrees of freedom) was selected. Model fit and statistical criteria were used with substantive interpretability to evaluate each model.

For Bayesian SEM, Models 1 and 2 were examined by specifying a series of priors (i.e., prior parameter distributions). Bayesian SEM can include two types of priors: non-informative and informative. They differ in the degree of specification imposed on the models, with informative priors specifying the particular distribution of parameters, as compared to non-informative priors, which do not specify such particular distributions. Our analyses using conventional and Bayesian SEM were also different in terms of cross-loadings and residual correlations. As mentioned above, Bayesian SEM can include not only major loadings but also cross-loadings and residual correlations that have small, non-major effects on the model (expressed as a dotted line) by specifying informative priors. Specifying approximate zeros is more realistic than specifying exact zeros (e.g., de Bondt and van Petegem, 2015).

In Model 1a, non-informative priors were specified for factor loadings, with normally distributed priors with a mean of zero and infinite variance, and for observed variable variances, with inverse gamma distribution priors with infinite means and variances. In Model 2a, non-informative priors were additionally specified for factor (co)variance(s), with an inverse-Wishart distribution prior with a mean of zero and the degree of freedom of the model. These specifications were the software-default settings of Mplus. In Model 2b, informative priors were additionally specified for cross-loadings, with normally distributed priors with a mean of zero and a variance of 0.01. A variance of 0.01 results in 95% cross-loading limits of ± 0.20 (Muthén and Asparouhov, 2012). This means that factor loadings vary in size between ± 0.20, although their means are zero. For example, this permitted the modeling of the small, non-major effects of vocabulary depth on vocabulary size. In Models 1c and 2c, informative priors were additionally specified for residual covariances, with inverse-Wishart distribution priors with a mean of zero and the degree of freedom of the model.

Model convergence was judged using (a) potential scale reduction (PSR) values and (b) Bayesian posterior parameter trace plots showing little change at each iteration. For (a), the value should be 1.0 at convergence, but values of less than 1.1 are considered acceptable. Model fit was assessed using posterior predictive *p* values of near 0.5, with 95% confidence intervals (CIs) that should be symmetric and center around 0. The models were compared using the deviance information criterion and Bayesian information criterion. For details on these criteria for convergence, model fit, and model comparison (see Muthén and Asparouhov, 2012; de Bondt and van Petegem, 2015; Norouzian et al., 2018).

## Results

### Descriptive Statistics

Table 2 shows that internal consistency was high for all vocabulary tests (α = 0.74–0.88), except for the Word Association Test (α = 0.56). Pearson product-moment correlations between size and depth indicators ranged from small to moderate (0.297–0.793), which were lower than the criterion for concerns about multicollinearity (*r*≥ 0.9; Tabachnick and Fidell, 2014). Note that correlations between Polysemy 1 and Polysemy 2 and between Association and Collocation were not high (*r* = 0.498, 0.297). This suggests that these tests measure rather different, marginally overlapping constructs.

**TABLE 2:** Summary statistics for and correlations between the vocabulary tests.

	***M***	***SD***	**Skewness**	**Kurtosis**	**α**	**Full mark**	**(1)**	**(1a)**	**(1b)**	**(1c)**	**(2)**	**(3)**	**(4)**
(1) Size	25.25	8.57	0.00	–1.07	0.91	40	–						
(1a) Size123	11.61	2.84	–0.65	–0.51	0.74	15	0.891 [0.862, 0.913]	–					
(1b) Size456	8.37	3.86	0.10	–0.96	0.83	15	0.942 [0.927, 0.955]	0.743 [0.683, 0.794]	–				
(1c) Size78	5.29	2.64	0.10	–0.98	0.82	10	0.906 [0.881, 0.926]	0.724 [0.660, 0.778]	0.793 [0.742, 0.834]	–			
(2) Association	16.82	3.52	–0.03	–0.27	0.56	30	0.530 [0.435, 0.613]	0.485 [0.385, 0.573]	0.486 [0.386, 0.574]	0.486 [0.386, 0.574]	–		
(3) Polysemy 1	11.39	4.25	0.03	–0.94	0.78	20	0.814 [0.768, 0.852]	0.721 [0.656, 0.775]	0.769 [0.713, 0.815]	0.739 [0.678, 0.790]	0.514 [0.418, 0.599]	–	
(4) Polysemy 2	8.56	4.69	–0.37	–0.67	0.88	17	0.504 [0.406, 0.590]	0.451 [0.348, 0.544]	0.466 [0.364, 0.557]	0.467 [0.365, 0.558]	0.351 [0.239, 0.454]	0.498 [0.399, 0.585]	–
(5) Collocation	10.83	4.06	–1.06	1.54	0.77	20	0.422 [0.315, 0.518]	0.359 [0.247, 0.461]	0.403 [0.295, 0.501]	0.392 [0.283, 0.492]	0.297 [0.181, 0.405]	0.453 [0.350, 0.546]	0.368 [0.257, 0.469]

*N = 255. Size123 = size with 1,000–3,000 levels; Size456 = size with 4,000–6,000 levels; Size78 = size with 7,000 and 8,000 levels. [] = 95% confidence intervals (CIs). All correlations were statistically significant, p < 0.01. (1) Size was a composite of (1a) Size123, (1b) Size456, and (1c) Size78 and was not analyzed using not analyzed using structural equation modeling (SEM). Instead, these three variable were analyzed using SEM.*

While detailed analysis is conducted using SEM, results of simple correlations between size and depth are reported for the sake of comparison with previous studies. Correlations of Size with Association, Polysemy 2, and Collocation were moderate, ranging from 0.422 to 0.530. These correlations were considered similar because their 95% CIs overlapped with each other. The correlation between Size and Polysemy 1 was strong (*r* = 0.814), and its 95% CI did not overlap with the CIs of the correlations of Size with Association, Polysemy 2, or Collocation. Differences between Polysemy 1 and 2 in relation to their correlations with Size arose mainly due to minor differences between constructs. Polysemy 1 assessed the knowledge of primary and secondary meanings, whereas Polysemy 2 assessed the knowledge of only the secondary meaning.

### Conventional SEM

As seen in Table 3, Models 1 and 2 fit the data well (e.g., SRMR = 0.024 and 0.018, respectively). A comparison of these two models revealed Model 2 to be the best model to represent the structure of vocabulary knowledge for the current data, as shown by a lower AIC (4,113.674 vs. 4,107.574 for Models 1 and 2, respectively) and the significant result produced by a chi-square difference test (the chi-square difference between the two models was 5.806, exceeding the critical value of 3.841 at *p* < 0.05). The standardized parameter estimates [see the column “Conventional SEM” (Model 2) in Table 4] show that each vocabulary factor was, overall, well explained by the tests (vocabulary size: 0.832 for Size123 to 0.899 for Size456; vocabulary depth 0.496 for Collocation to 0.901 for Polysemy 1). The vocabulary size and depth factors were highly correlated (*r* = 0.945). Thus, size and depth are considered to be separate but closely related.

**TABLE 3:** Results from maximum likelihood estimation with robust standard errors estimation for the single-factor and the correlated two-factor models.

		**χ^2^**	***df***	***p***	**CFI**	**RMSEA [90% CI]**	**SRMR**	**AIC**	**Fit?**	**Best-fitting model?**
	Criteria	The smaller, the better	–	> 0.05	>0.90	< 0.08	<0.08	The smaller, the better	–	–
Model 1	Single-factor	16.153	14	0.304	0.997	0.023 [0.000, 0.068]	0.024	4,113.674	Yes	
Model 2	Correlated two-factor	8.391	13	0.817	1.000	< 0.001 [0.000, 0.039]	0.018	4,107.574	Yes	Yes

**N = 255. CFI, comparative fit index; RMSEA, root mean square error of approximation; SRMR, standardized root mean square residual; AIC, Akaike information criterion. The scaling correction factors for maximum likelihood estimation with robust standard errors estimation (MLR) were 1.0116 and 0.9821 for Models 1 and 2, respectively.**

**TABLE 4:** Parameter estimate of the correlated two-factor model.

	**Conventional SEM (Model 2)**				**Bayesian SEM (Model 2b)**			
		
	**Size**		**Depth**		**Size**		**Depth**	
				
	**Unstandardized**	**Standardized**	**Unstandardized**	**Standardized**	**Unstandardized**	**Standardized**	**Unstandardized**	**Standardized**
Size123	1.000	0.832			**1.000**	**0.817**	0.026	0.014
Size456	1.080	0.899			**1.102**	**0.900**	−0.002	−0.001
Size78	1.051	0.875			**1.074**	**0.877**	−0.007	−0.003
Association			1.000	0.582	0.024	0.019	**1.000**	**0.559**
Polysemy 1			1.548	0.901	0.027	0.022	**1.570**	**0.877**
Polysemy 2			0.971	0.565	−0.004	−0.003	**1.018**	**0.568**
Collocation			0.853	0.496	−0.028	−0.022	**0.929**	**0.518**
Correlation and covariance between size and depth	0.458	0.945			0.432	0.943		

**N = 255. SEM = structural equation modeling. All unstandardized parameters except for those fixed to 1 for identification were statistically significant. For Bayesian SEM, values in bold indicate hypothesized major loadings.**

### Bayesian SEM

Table 5 shows the results for Bayesian estimation. Models 1a, 2a, and 2b converged, whereas Models 1c and 2c did not. For example, Model 1a had a PSR value of 1.001, which was very close to 1.0 and less than 1.1. Bayesian posterior parameter trace plots, although not reported here, showed a stable, horizontal band for the parameter in question. These results suggest the convergence of the parameters in the model. On the other hand, Models 1c and 2c failed to converge. For example, Model 1c had a PRS value of 5.463, which considerably exceeded 1.1. Bayesian posterior parameter trace plots, although not reported here, showed a widely fluctuating, horizontal band for the parameter in question. These results suggest that Models 1c and 2c displayed poor convergence for their parameters.

**TABLE 5:** Results from Bayesian estimation for the single-factor and the correlated two-factor models.

			**Convergence**			**Model fit**					
			**PSR**	**BPPTPs**	**Converged?**	**PP *p***	**95% CrI of PP *p***	**DIC**	**BIC**	**Fit?**	**Best-fitting?**
		Criteria	1.0; 1.1 or less	Changes little on each iteration	–	Near 0.5	Symmetric, centering around 0	The smaller, the better	The smaller, the better	–	–
Model 1a	Single-factor	Non-informative priors	1.001	Changes little on each iteration	Yes	0.392	−18.620, 24.364	4,113.585	4,188.459	Yes	
Model 1b	Single-factor	Informative priors (SVPs for CLs)	This model was not tested, since it had only one factor and cross-loadings were not applicable accordingly.								
Model 1c	Single-factor	Informative priors (SVPs for RVs)	5.463	Changes much on each iteration	No	0.535	-24.541, 22.141	4,123.138	4,295.869	–	
Model 2a	Correlated two-factor	Non-informative priors	1.007	Changes little on each iteration	Yes	0.661	-25.924, 16.367	4,106.968	4,186.052	Yes	
Model 2b	Correlated two-factor	Informative priors (SVPs for CLs)	1.001	Changes little on each iteration	Yes	0.670	-26.900, 16.065	4,103.706	4,225.306	Yes	Yes
Model 2c	Correlated two-factor	Informative priors (SVPs for CLs and RVs)	5.493	Changes much on each iteration	No	0.450	-22.548, 24.308	4,123.121	4,344.999	–	

*N = 255. PSR, potential scale reduction; BPPTPs, Bayesian posterior parameter trace plots; PP p, posterior predictive p-value; CrI, credibility interval; DIC, deviance information criterion; BIC, Bayesian information criterion; SVPs, small-variance priors; CLs, cross-loadings; RVs, residual variances. Results are based on 10,000 iterations. Non-convergent model were further tested with 100,000 iterations. Models 1c and 2c still failed to meet convergence criterion in terms of PSR and BPPTPs.*

A comparison of converged models – Models 1a, 2a, and 2b – revealed that Models 2a and 2b were statistically equally likely, which is supported by similar values for model fit indices. Nevertheless, Model 2b was considered to best represent the structure of vocabulary knowledge for the current data, given that it was sensible to specify small-variance cross-loadings: The role of vocabulary size was very small but not zero in the vocabulary depth tests, and the role of vocabulary depth was, likewise, very small but not zero in the size test. The standardized parameter estimates [see the column “Bayesian SEM” (Model 2b) in Table 4] show that each skill factor was, overall, well explained by the tests (vocabulary size: 0.817 for Size123 to 0.900 for Size456; vocabulary depth: 0.518 for Collocation to 0.877 for Polysemy 1). The vocabulary size and depth factors were highly correlated (*r* = 0.943). Cross-loadings were very close to zero. This shows that the size and depth measures were successful in assessing separate constructs. In conclusion, as with the results from conventional SEM, Bayesian SEM showed that both size and depth are separately modeled but closely related.

## Discussion

To examine the relationship between vocabulary size and depth for low- to intermediate-level Japanese learners of English using conventional and Bayesian SEM, the following research question was addressed: Which factor structure of the size and depth of vocabulary knowledge explains the data better, a single-factor or a correlated two-factor model? Vocabulary knowledge was modeled as a single factor (i.e., vocabulary size and depth as one entity) and as two correlated factors (i.e., vocabulary size and depth as separately conceptualized), and model fit was compared. The results of conventional SEM showed that the correlated two-factor model best explained the data. The results from Bayesian SEM showed that the best-fitting model was the correlated two-factor model with very small cross-loadings. For both models, vocabulary size and depth factors were highly correlated (*r* = 0.945 for conventional SEM and 0.943 for Bayesian SEM). Thus, both size and depth are closely related yet two separate constructs. It is worth recalling that the structure of language ability has been an important research area, and the current results of having two lexical components strongly correlated to each other would add to the existing literature of the multicomponential nature of language ability.

The strong relationship between size and depth is consistent with a previous study using SEM (Vafaee, 2016). The adoption of the correlated two-factor model over the single-factor model in the current study as well as in Vafaee (2016) suggests that, even with very high correlations of 0.9 or above (*r* = 0.943–0.945 in the current study; *r* = 0.94 in Vafaee, 2016), distinguishing the two factors better explains the data than analyzing them as one factor. This means that size and depth should be distinguished conceptually and statistically. In other words, a person who knows more words (i.e., vocabulary size) tends to have a deeper vocabulary knowledge (i.e., vocabulary depth), but researchers and practitioners should still consider size and depth separately.

It should be recalled that Vafaee (2016) and the current study differ in (a) the measures used to assess size and depth, (b) the aspects assessed by depth tests, and (c) the participants’ L2 proficiency levels and their L1: Vafaee (2016) used an aural size test of meaning recall and an aural depth test (the WAF) of selecting synonyms and collocations, whereas we used a written size of form recognition and four depth tests focusing on association, polysemy, and collocation. Vafaee’s (2016) participants were Persian learners of English at lower-intermediate to advanced levels, whereas the current study’s participants were Japanese learners of English at low to intermediate levels. These differences may suggest some degree of generalizability regarding the factor structure of size and depth.

However, both studies involved L2 adult learners of English as a foreign language and multiple-choice formats of assessing size and depth. The relatively similar type of learners and the use of the same formats for measuring size and depth may have produced similar results across studies. Additionally, there was an overlap in the assessed depth aspects, with collocation tested in both studies. Synonym in Vafaee (2016) and “association and polysemy” in the current study are also related to meaning and are more similar to size (defined as knowledge of a word form and a primary meaning) than are other aspects of depth such as word parts. Vafaee (2016) and the current study showed that size and depth can be separately modeled, even when depth is operationalized as something similar to size. Thus, we can assume that when depth is operationalized as something more different from size, size and depth can also be separately modeled, with different degrees of correlations expected between size and depth, but this requires empirical research. Note that the results in the final models suggest that association, polysemy, and collocation measures primarily assess depth, not size: In Model 2 in conventional SEM, the three depth aspects loaded on the depth factor only (β = 0.496–0.901); in Model 2b in Bayesian SEM, the three depth aspects loaded on the depth factor to a large degree (β = 0.518–0.877) and on the size factor to negligible degrees (β = -0.022–0.022).

The use of SEM can help clarify the latent relationships between size and depth, but it is usually difficult to compare the SEM results with previous studies using simple correlation. We suggest three ways for effectively using previous study results. First, it is possible to model relationships using SEM when previous studies report means and *SD*s of the variables, and all correlations between them (see In’nami and Koizumi, 2010, 2012b; Vafaee and Kachinske, 2019), to examine how the latent factors of size and depth are correlated. However, this is often difficult because of the lack of reports of necessary statistics (Larson-Hall and Plonsky, 2015; see also Kline, 2016, p. 65, for cases where summary statistics are not enough and raw data are required) and the research design of previous studies. For example, previous studies often used only one measure of size, and it is, therefore, difficult to model it as a size factor.

The second and third methods use simple correlations and other descriptive statistics. Simple comparisons between correlation coefficients are not very productive because they are often affected by measurement error and sample size. In the second method, using reliability coefficients reported by previous studies, researchers can estimate the strength of correlation coefficients in the case of perfect test reliabilities (i.e., with no measurement error), using a formula for correcting for attenuation (Glass and Hopkins, 1996):

(Correlation coefficient between the first and second tests)/(√[reliability coefficient of the first test] × √[reliability coefficient of the second test]).

For example, the correlation between Size and Collocation was *r* = 0.422, with the reliability of the two tests being α = 0.91 and 0.77, in the current study. The corrected correlation is 0.575 (i.e., 0.422/[√0.91 × √0.77]), which is higher than the original value, 0.422. Thus, the use of this method of correcting for attenuation allows researchers to examine relationships while at the same time accounting for measurement error. This concept is similar to the one used in SEM (Hancock and Schoonen, 2015). The second method is relatively simple but is often difficult to execute because reliability results for all the variables are not always reported (Larson-Hall and Plonsky, 2015). Table 6 summarizes the previous studies reviewed in the *Literature Review* as well as the current study. It shows that the three studies using simple correlations (i.e., Schmitt and Meara, 1997; Vermeer, 2001; Akbarian, 2010) did not report reliability sufficiently, and this hampers the use of the second method.

**TABLE 6:** Reliability and confidence intervals of correlation coefficients between size and depth in previous studies.

	***n***	**Reliability**	**Original *r* reported in the article**	**95% CI of *r***
Vermeer (2001)	25	Size: NR Depth: α = 0.85	0.72–0.76	0.455, 0.868 0.522, 0.888
Akbarian (2010)	112	Size and depth: NR	0.864	0.808, 0.904
Schmitt and Meara (1997)	88	Size and depth: NR	0.27–0.62	0.065, 0.453 0.472, 0.734
Vafaee (2016)	263	Size: α = 0.67–0.84 Depth: α = 0.92–0.93	0.64–0.77	0.563, 0.706 0.716, 0.815
Current study	255	Size: α = 0.91 Depth: α = 0.56–0.77	0.422–0.814	0.315, 0.518 0.768, 0.852

**NR, not reported. We used the website for calculating a “confidence interval for an observed (Pearson) correlation coefficient” (http://vassarstats.net/rho.html).**

The third method uses 95% CIs of correlation coefficients. The use of CIs allows researchers to view sampling statistics (i.e., correlation coefficients) as values that fluctuate. In fact, researchers are encouraged to report CIs along with the point estimates (e.g., means and effect sizes; Norris et al., 2015; American Psychological Association, 2020). Although CIs are not always reported (Larson-Hall and Plonsky, 2015), CIs of correlation coefficients can be calculated using free online calculators^3^. The information of 95% CIs shows that if similar studies are conducted many times, 95% of those CIs will capture the population correlation. If 20 studies are conducted on the same topic, 19 (20^∗^0.95) of those CIs will capture the population correlation. Considering CIs along with the point estimates allows researchers to interpret results more accurately. For example, correlations between Size and Collocation (*r* = 0.422) and between Size and Association (*r* = 0.530) appear different, but in fact they are not, considering the substantial overlap of their 95% CIs (0.315 and 0.518 for the former; 0.435 and 0.613 for the latter; see Table 2). Table 6 shows 95% CIs of the correlations of previous studies and the current study, suggesting a similarity of relationships. The results can be quite a contrast when compared with the results using correlation coefficients only. For example, Vermeer (2001) has a wide CI (e.g., 0.455, 0.868) because the number of participants is small (*n* = 25), and the lower end of the CI (0.455) is very close to the upper end of the CI in Schmitt and Meara (1997; 0.065, 0.453). Schmitt (2014) suggested that different degrees of correlations between size and depth may be derived from different measures and different depth aspects measured, but different sample sizes and resulting measurement error may also be other factors. Future research should consider using the abovementioned three methods, especially CIs, for comparing previous studies. Additionally, other methods that would provide more precise estimates would be (a) a bootstrapping method (McLean et al., 2020), which is useful when primary data are available, and (b) meta-analysis, which can systematically integrate previous studies while taking sample size and measurement error into account (e.g., Plonsky and Oswald, 2015; In’nami et al., 2020). When researchers obtain a matrix of meta-analyzed correlation coefficients through (b), they can more rigorously examine relationships of size and depth using meta-analytic SEM (Cheung, 2015).

From a methodological viewpoint, it is important to note that for the best-fitting correlated two-factor model, the vocabulary size and depth factors were highly correlated (*r* = 0.945 for conventional SEM and 0.943 for Bayesian SEM with cross-loadings). Recall that Bayesian SEM was used in the current study to more flexibly examine the factor structure of vocabulary size and depth by specifying cross-loadings and residual correlations. Obtaining similar factor structures with similarly high correlations between vocabulary size and depth for conventional and Bayesian SEM indicates the robustness of such structures and correlations. This was revealed only after comparing the findings from conventional and Bayesian SEM approaches. It should be noted that this does not mean that conventional SEM is sufficient and the use of Bayesian SEM is redundant. Bayesian SEM allows for more varied specifications of parameter distribution that were not used in the current study. This advantage of Bayesian SEM should be best employed in future vocabulary studies.

## Conclusion

We examined the factor structure of the size and depth of vocabulary knowledge with five tests focusing on size and depth (association, polysemy, and collocation). We found that a correlated two-factor model explained the data better than a single-factor model. As introduced in the *Literature Review*, Schmitt (2014) asked, “Do size and depth behave as separate constructs,” “or are they essentially the same construct?” (p. 941). Our answer to these questions based on the findings of the current study is affirmative for the first question: Size and depth can be considered separate constructs, even when depth is measured by tests assessing aspects related to meaning and more similar to size.

Our study highlights the importance of distinguishing size and depth as two correlated but separate aspects of L2 vocabulary knowledge. This finding has implications for practice and theory. First, for L2 vocabulary assessment, if the purpose of the tests is to assess overall vocabulary knowledge, both size and depth should be included in tests to minimize construct underrepresentation of vocabulary knowledge. Given that vocabulary knowledge consists of size and depth, the inclusion of both aspects should maximize and best represent the construct of vocabulary knowledge (see Tseng and Schmitt, 2008; Zhang, 2012; Koizumi and In’nami, 2013; Vafaee and Suzuki, 2020; for examples). Further, in scoring and interpreting tests that include size and depth, separate scoring and interpretation of size and depth is justified, even though the two sections can be combined into a total score, given the high correlation between size and depth. When test developers or users conduct a validation study of their vocabulary tests that include size and depth, they should consider modeling these two when possible so as to examine the factor structure of their tests. For example, they can model a size factor using different frequency band scores and a depth factor using different section scores assessing depth. Moreover, in L2 instruction, teachers need to consider enhancing both size and depth as possible instructional goals and design, and allocate tasks for increasing these lexical components in a course (see Nation, 2013; Webb and Nation, 2017; Newton, 2020, for suggestions on effective learning and teaching in class).

Second, for theory building, the use of SEM for model construction and testing is helpful in empirically investigating relationships by considering measurement error. This allows researchers to examine the relationship between constructs of interest while separately estimating the impact of measurement error. This is how the close relationship (more than *r* = 0.9) between vocabulary size and depth was revealed in the current study. It should be recalled that simple correlations between size and depth measures were not that strong (0.422–0.793), which clearly shows one of the strengths of SEM. Taking this step further, minor cross-loadings and minor residual correlations were specified by Bayesian SEM. This would not have been possible with conventional SEM. These features of Bayesian SEM should help construct more realistic models to test specific hypotheses.

We have expanded previous studies and examined three depth aspects (association, polysemy, and collocation) by using separate tests, not the WAF. However, our results may be limited, as we targeted only Japanese adult learners of English at low to intermediate levels. What is needed are studies with different types of learners of diverse L1 and L2.

Further, we used only one measure of size (with three indicators at different levels of word frequency) and four measures of depth. There are other aspects that are important but were not examined (e.g., spoken forms, word parts, grammatical functions), and future research should include measures of size and depth by using various instruments (e.g., Godfroid, 2020, for offline and online measures to cover Nation, 2020, vocabulary elements) to examine their relationships. Tests should be developed, and test validation should be conducted, by following the principles summarized by Schmitt et al. (2020).

Specifically, to improve measures in the current study, the following three points are stated. First, we used only the multiple-choice format. A problem with this format is that it allows guesswork and overestimates the scores. Gyllstad et al. (2015) showed that takers of multiple-choice tests make educated (e.g., using the knowledge of word family) as well as blind guesses. For a more valid measurement, recall and other formats should also be employed. Second, the vocabulary size test may have had a low sampling rate. According to Gyllstad et al. (2015), 30 words (taken from a pool of 1,000 words) function with greater precision than 10 words in a vocabulary size test. In the current test, we included a maximum of 40 items in the test battery (a total of 130 items to be attempted in 80 min) keeping in mind the issue of test takers’ concentration. Future research should consider how to manage the balance between the assessment need for including more items – so as to have a representative sample of vocabulary – and the practical need to reduce the items (Gyllstad, 2020). Third, some items in depth tests may need improvement. In particular, the collocation test should use a standard recently employed for selecting collocations, such as a minimum frequency of 10–50 in a corpus and an MI score of 3.00 or more (Kremmel and Schmitt, 2016; Nguyen and Webb, 2017).

In order to provide researchers with a comprehensive picture of relationships between size and depth, the following are required: a wider range of participants, and size and depth measures with larger item size, better quality, and wider focus. Comparisons of size–depth relationships across different L2 proficiency groups using multi-sample SEM (In’nami and Koizumi, 2012a; Zhang et al., 2014) would further elucidate intricate relationships. It would also be possible to model various aspects of depth separately as latent factors to specify more precise models of size and depth (see Stewart et al., 2013, for an attempt at modeling spoken vocabulary knowledge, polysemous word knowledge, and contextual word knowledge). The current study’s insight into the highly correlated but distinctive nature of size and depth should help researchers advance the understanding of the structure of vocabulary knowledge.

## Data Availability Statement

The datasets generated for this study are available on request to the corresponding author.

## Ethics Statement

Ethical review and approval was not required for the study on human participants in accordance with the local legislation and institutional requirements. Written informed consent for participation was not required for this study in accordance with the national legislation and the institutional requirements.

## Author Contributions

RK conceptualized, designed, and conducted the study, organized the data, performed the conventional SEM, and wrote the first draft of the manuscript and revised it throughout. YI performed the Bayesian SEM, revised the manuscript and added sections to the manuscript. RK and YI contributed to manuscript revision and read and approved the submitted version.

## Conflict of Interest

The authors declare that the research was conducted in the absence of any commercial or financial relationships that could be construed as a potential conflict of interest.

## Footnotes

1 http://language.sakura.ne.jp/s/voc.html

2 https://www.english-corpora.org/corpora.asp

3 https://www.psyctc.org/stats/R/CI_correln1.html; http://vassarstats.net/rho.html

## References

[B1] Akase M. (2005). The roles of breadth and depth of vocabulary knowledge in EFL reading comprehension: with a focus on English major students. *Ann. Rev. English Lang. Educ. Jp.* 16 141–150. 10.20581/arele.16.0_141

[B2] Akbarian I. (2010). The relationship between vocabulary size and depth for ESP/EAP learners. *System* 38 391–401. 10.1016/j.system.2010.06.013

[B3] American Psychological Association (2020). *Publication Manual of the American Psychological Association*, 7th Edn Washington, DC: American Psychological Association, 10.1037/0000165-000

[B4] Anderson R. C. Freebody P. (1981). “Vocabulary knowledge,” in *Comprehension and Teaching: Research Reviews*, ed. Guthrie J. T. (Newark, DE: International Reading Association), 77–117.

[B5] Arbuckle J. L. Wothke W. (1995). *Amos 4.0 User’s Guide.* Chicago: SmallWaters Corporation.

[B6] Bachman L. F. Palmer A. S. (1983). “The construct validation of the FSI oral interview,” in *Issues in Language Testing Research*, ed. Oller J. W. Jr. (Rowley, MA: Newbury House), 154–169.

[B7] Bae J. Bentler P. M. Lee Y.-S. (2016). On the role of content in writing assessment. *Lang. Assess. Q.* 13 302–328. 10.1080/15434303.2016.1246552

[B8] Chapelle C. A. (1998). “Construct definition and validity inquiry in SLA research,” in *Interfaces Between Second Language Acquisition and Language Testing Research*, eds Bachman L. F. Cohen A. D. (Cambridge: Cambridge University Press), 32–70. 10.1017/cbo9781139524711.004

[B9] Chapelle C. A. Enright M. K. Jamieson J. M. (2008). *Building a Validity Argument for the Test of English as a Foreign Language^TM^.* New York, NY: Routledge.

[B10] Cheung M. W.-L. (2015). *Meta-Analysis: A Structural Equation Modeling Approach.* West Sussex: John Wiley & Sons.

[B11] Daller H. Milton J. Treffers-Daller J. (2007). “Editors’ introduction: conventions, terminology and an overview of the book,” in *Modelling and Assessing Vocabulary Knowledge*, eds Daller H. Milton J. Treffers-Daller J. (Cambridge, MA: Cambridge University Press), 1–32.

[B12] de Bondt N. van Petegem P. (2015). Psychometric evaluation of the Overexcitability Questionnaire–Two applying Bayesian structural equation modeling (BSEM) and multiple-group BSEM-based alignment with approximate measurement invariance. *Front. Psychol.* 6:1963 10.3389/fpsyg.2015.01963 PMC4689874 26733931

[B13] Educational Testing Service (2019). *2018 Report on Test Takers Worldwide–TOEIC^®^ Listening & Reading Test.* Princeton, NJ: Author.

[B14] Farhady H. (1983). “On the plausibility of the unitary language proficiency factor,” in *Issues in Language Testing Research*, ed. Oller J. W. Jr. (Rowley, MA: Newbury House), 11–28.

[B15] Glass G. V. Hopkins K. D. (1996). *Statistical Methods in Education and Psychology*, 3rd Edn Boston, MA: Allyn & Bacon.

[B16] Godfroid A. (2020). “Sensitive measures of vocabulary knowledge and processing: expanding Nation’s framework,” in *The Routledge Handbook of Vocabulary Studies*, ed. Webb S. (London: Routledge), 433–453. 10.4324/9780429291586-28

[B17] Gorfein D. A. Viviani J. M. Leddo J. (1982). Norms as a tool for the study of homography. *Mem. Cogn.* 10 503–509. 10.3758/BF03197654 7176912

[B18] Gyllstad H. (2020). “Measuring knowledge of multiword items,” in *The Routledge Handbook of Vocabulary Studies*, ed. Webb S. (London: Routledge), 387–405. 10.4324/9780429291586-25

[B19] Gyllstad H. Vilkaitė L. Schmitt N. (2015). Assessing vocabulary size through multiple-choice formats: issues with guessing and sampling rates. *Int. J. Appl. Linguist.* 166 278–306. 10.1075/itl.166.2.04gyl

[B20] Hancock G. R. Schoonen R. (2015). Structural equation modeling: possibilities for language learning researchers. *Lang. Learn.* 65(Suppl. 1), 160–184. 10.1111/lang.12116

[B21] Henriksen B. (1999). Three dimensions of vocabulary development. *Stud. Sec. Lang. Acquis.* 21 303–317. 10.1017/S0272263199002089

[B22] Hu L.-T. Bentler P. M. (1999). Cutoff criteria for fit indexes in covariance structure analysis. *Struc. Equ. Model.* 6 1–55. 10.1080/10705519909540118

[B23] In’nami Y. Koizumi R. (2010). Can structural equation models in second language testing and learning research be successfully replicated? *Int. J. Test.* 10 262–273. 10.1080/15305058.2010.482219

[B24] In’nami Y. Koizumi R. (2011). Structural equation modeling in language testing and learning research: a review. *Lang. Assess. Q.* 8 250–276. 10.1080/15434303.2011.565844

[B25] In’nami Y. Koizumi R. (2012a). Factor structure of the revised TOEIC^®^ test: a multiple-sample analysis. *Lang. Test.* 29 131–152. 10.1177/0265532211413444

[B26] In’nami Y. Koizumi R. (2012b). “Reproduction of structural equation models in second language testing and learning research,” *Reports of 2011 Studies in Japan Association for Language Education and Technology*, Kansai Chapter, Methodology Special Interest Groups (SIG), Vol 2 15–40.

[B27] In’nami Y. Koizumi R. Tomita Y. (2020). “Meta-analysis in applied linguistics,” in *The Routledge Handbook of Research Methods in Applied Linguistics*, eds McKinley J. Rose H. (New York, NY: Routledge), 240–252. 10.4324/9780367824471-21

[B28] Ishii T. Schmitt N. (2009). Developing an integrated diagnostic test of vocabulary size and depth. *RELC J.* 40 5–22. 10.1177/0033688208101452

[B29] JACET Basic Word Revision Committee (2016). *Nihonjin Daigakusei yo kihon goi shin JACET8000 [The new JACET list of 8000 Basic Words].* Tokyo: Kirihara Shoten.

[B30] JACET Basic Word Revision Committee Ed. (2003). *JACET List of 8000 Basic Words.* Tokyo: Author.

[B31] Kane M. T. (2013). Validating the interpretations and uses of test scores. *J. Educ. Measure.* 50 1–73. 10.1111/jedm.12000

[B32] Kieffer M. Lesaux N. (2012). Knowledge of words, knowledge about words: dimensions of vocabulary in first and second language learners in sixth grade. *Read. Writ.* 25 347–373. 10.1007/s11145-010-9272-9

[B33] Kline R. B. (2016). *Principles and Practice of Structural Equation Modeling*, 4th Edn New York, NY: Guilford.

[B34] Koizumi R. In’nami Y. (2013). Vocabulary knowledge and speaking proficiency among second language learners from novice to intermediate levels. *J. Lang. Teach. Res.* 4 900–913. 10.4304/jltr.4.5.900-913

[B35] Korkmaz S. Goksuluk D. Zararsiz G. (2019). *MVN.* Available online at: https://cran.r-project.org/web/packages/MVN/MVN.pdf

[B36] Kremmel B. Schmitt N. (2016). Interpreting vocabulary test scores: what do various item formats tell us about learners’ ability to employ words? *Lang. Assess. Q.* 13 377–392. 10.1080/15434303.2016.1237516

[B37] Larson-Hall J. Plonsky L. (2015). Reporting and interpreting quantitative research findings: what gets reported and recommendations for the field. *Lang. Learn.* 65(Suppl. 1), 127–159. 10.1111/lang.12115

[B38] Laufer B. Elder C. Hill K. Congdon P. (2004). Size and strength: do we need both to measure vocabulary knowledge? *Lang. Test.* 21 202–226. 10.1191/0265532204lt277oa

[B39] Little T. D. Cunningham W. A. Shahar G. (2002). To parcel or not to parcel: exploring the question, weighing the merits. *Struc. Equ. Model.* 9 151–173. 10.1207/S15328007SEM0902_1

[B40] Little T. D. Rhemtulla M. Gibson K. Schoemann A. M. (2013). Why the items versus parcels controversy needn’t be one. *Psychol. Methods* 18 285–300. 10.1037/a0033266 23834418 PMC3909043

[B41] McLean S. Stewart J. Batty A. O. (2020). Predicting L2 reading proficiency with modalities of vocabulary knowledge: a bootstrapping approach. *Lang. Test.* 10.1177/0265532219898380

[B42] Meade A. W. Kroustalils C. W. (2006). Problems with item parceling for confirmatory factor analytic tests of measurement invariance. *Organ. Res. Methods* 9 369–403. 10.1177/1094428105283384

[B43] Meara P. (2005). “Designing vocabulary tests for English, Spanish, and other languages,” in *The Dynamics of Language Use*, eds Butler C. S. Gómez-González M. A. Doval-Suárez S. M. (Amsterdam: John Benjamins), 271–285. 10.1075/pbns.140.19mea

[B44] Milton J. (2009). *Measuring Second Language Vocabulary Acquisition.* Bristol: Multilingual Matters.

[B45] Mochizuki M. Aizawa K. (2000). An affix acquisition order for EFL learners: an exploratory study. *System* 28 291–304. 10.1016/s0346-251x(00)00013-0

[B46] Mochizuki M. Uemura T. Aizawa K. Sugimori N. Ishikawa S. Iso T. (2014). *Goichishiki Sokutei Niyoru Eigo Noryoku no suitei: Goi saizu, kosei, akusesu sokudo no kantenkara [Estimation of English ability using measures of vocabulary size, organization, and access speed of vocabulary knowledge]. Report of the Grant-in-Aid for Scientific Research (B)(2010-2013), Supported by Japan Society for the Promotion of Science. Project No. 22320110.* Tokyo: Japan Society for the Promotion of Science Available online at: http://mochvocab.sakura.ne.jp/img/file6.pdf

[B47] Mplus (2019). *Chi-square Difference Testing Using the Satorra-Bentler Scaled Chi-Square.* Los Angeles, CA: Mplus.

[B48] Muthén B. Asparouhov T. (2012). Bayesian structural equation modeling: a more flexible representation of substantive theory. *Psychol. Methods* 17 313–335. 10.1037/a0026802 22962886

[B49] Muthén and Muthén (1998–2019). *Mplus (Version 8.3) [Computer software].* Los Angeles, CA: Muthén & Muthén.

[B50] Nation I. S. P. (2013). *Learning Vocabulary in Another Language*, 2nd Edn Cambridge, MA: Cambridge University Press.

[B51] Nation I. S. P. Beglar D. (2007). A vocabulary size test. *Lang. Teach.* 31 9–13.

[B52] Nation I. S. P. Webb S. (2011). *Researching and Analyzing Vocabulary.* Boston, MA: Heinle, Cengage Learning.

[B53] Nation P. (1983). Testing and teaching vocabulary. *Guidelines* 5 12–25.

[B54] Nation P. (2020). “The different aspects of vocabulary knowledge,” in *The Routledge Handbook of Vocabulary Studies*, ed. Webb S. (London: Routledge), 15–29. 10.4324/9780429291586-2

[B55] Nation P. Sorell J. (2016). “Corpus selection and design,” in *Making and using Word Lists for Language Learning and Testing*, ed. Nation I. S. P. (Amsterdam: John Benjamins), 95–105. 10.1075/z.208.10ch10

[B56] Newton J. (2020). “Approaches to learning vocabulary inside the classroom,” in *The Routledge Handbook of Vocabulary Studies*, ed. Webb S. (London: Routledge), 255–270. 10.4324/9780429291586-17

[B57] Nguyen T. M. H. Webb S. (2017). Examining second language receptive knowledge of collocation and factors that affect learning. *Lang. Teach. Res.* 21 298–320. 10.1177/1362168816639619

[B58] Noro T. (2002). The roles of depth and breadth of vocabulary knowledge in reading comprehension in EFL. *Ann. Rev. English Lang. Educ. Jpn.* 13 71–80. 10.20581/arele.13.0_71

[B59] Norouzian R. de Miranda M. Plonsky L. (2018). The Bayesian revolution in second language research: an applied approach. *Lang. Learn.* 68 1032–1075. 10.1111/lang.12310

[B60] Norris J. M. Plonsky L. Ross S. J. Schoonen R. (2015). Guidelines for reporting quantitative methods and results in primary research. *Lang. Learn.* 65 470–476. 10.1111/lang.12104

[B61] Nurweni A. Read J. (1999). The English vocabulary knowledge of Indonesian university students. *English Specific Purposes* 18 161–175. 10.1016/S0889-4906(98)00005-2

[B62] Ockey G. J. Choi I. (2015). Structural equation modeling reporting practices for language assessment. *Lang. Assess. Q.* 12 305–319. 10.1080/15434303.2015.1050101

[B63] Oller J. W. Jr. (1983). “Evidence for a general language proficiency factor: an expectancy grammar,” in *Issues in Language Testing Research*, ed. Oller J. W. Jr. (Rowley, MA: Newbury House), 3–10.

[B64] Plonsky L. Oswald F. L. (2015). “Meta-analyzing second language research,” in *Advancing Quantitative Methods in Second Language Research*, ed. Plonsky L. (New York, NY: Routledge), 106–128. 10.4324/9781315870908-6

[B65] Qian D. (2002). Investigating the relationship between vocabulary knowledge and academic reading performance: an assessment perspective. *Lang. Learn.* 52 513–536. 10.1111/1467-9922.00193

[B66] Qian D. D. Lin L. H. F. (2020). “The relationship between vocabulary knowledge and language proficiency,” in *The Routledge Handbook of Vocabulary Studies*, ed. Webb S. (London, UK: Routledge), 66–80. 10.4324/9780429291586-5

[B67] Read J. (1993). The development of a new measure of L2 vocabulary knowledge. *Lang. Test.* 10 355–371. 10.1177/026553229301000308

[B68] Read J. (1998). “Validating a test to measure depth of vocabulary knowledge,” in *Validation in language assessment*, ed. Kunnan A. J. (Mahwah, NJ: Lawrence Erlbaum Associates), 41–60. 10.4324/9780203053768

[B69] Read J. (2000). *Assessing Vocabulary.* Cambridge Cambridge University Press.

[B70] Read J. (2004). “Plumbing the depths: how should the construct of vocabulary be defined?,” in *Vocabulary in a Second Language: Selection, Acquisition, and Testing*, eds Bogaards P. Laufer B. (Amsterdam: John Benjamins), 209–227. 10.1075/lllt.10.15rea

[B71] Sawaki Y. (2007). Construct validation of analytic rating scales in a speaking assessment: reporting a score profile and a composite. *Lang. Test.* 24 355–390. 10.1177/0265532207077205

[B72] Sawaki Y. Sinharay S. (2017). Do the TOEFL iBT^®^ section scores provide value-added information to stakeholders? *Lang. Test.* 35 529–556. 10.1177/0265532217716731

[B73] Schmitt N. (2010). *Researching Vocabulary: A Vocabulary Research Manual.* Hampshire: Palgrave MacMillan.

[B74] Schmitt N. (2014). Size and depth of vocabulary knowledge: what the research shows. *Lang. Learn.* 64 913–951. 10.1111/lang.12077

[B75] Schmitt N. Meara P. (1997). Researching vocabulary through a word knowledge framework: word associations and verbal suffixes. *Stud. Sec. Lang. Acquis.* 20 17–36. 10.1017/S0272263197001022

[B76] Schmitt N. Nation P. Kremmel B. (2020). Moving the field of vocabulary assessment forward: the need for more rigorous test development and validation. *Lang. Teach.* 53 109–120. 10.1017/S0261444819000326

[B77] Schmitt N. Schmitt D. Clapham C. (2001). Developing and exploring the behaviour of two new versions of the vocabulary levels test. *Lang. Test.* 18 55–88. 10.1177/026553220101800103

[B78] Seto K. (2007). *Eigo Tagi Nettowaku Jiten. [Dictionary of English Lexical Polysemy].* Tokyo: Shogakukan.

[B79] Shimamoto T. (2005). Exploring lexical network systems of Japanese EFL learners through depth and breadth of word knowledge. *Ann. Rev. English Lang. Educ. Jpn.* 16 121–130. 10.20581/arele.16.0_121

[B80] Shin S.-K. (2005). Did they take the same test? Examinee language proficiency and the structure of language tests. *Lang. Test.* 22 31–57. 10.1191/0265532205lt296oa

[B81] Staehr L. S. (2009). Vocabulary knowledge and advanced listening comprehension in English as a foreign language. *Stud. Sec. Lang. Acquis.* 31 577–607. 10.1017/S0272263109990039

[B82] Stewart J. Fryer L. Gibson A. (2013). Assessing the dimensionality of three hypothesized sub-skills of L2 vocabulary proficiency. *JACET J.* 56 57–71.

[B83] Tabachnick B. G. Fidell L. S. (2014). *Using Multivariate Statistics*, 6th Edn Harlow: Pearson.

[B84] Tannenbaum K. R. (2008). *Relationships Between Measures of Word Knowledge and Reading Comprehension in Third- and Seventh-Grade Children.* Unpublished Ph.D. dissertation, Florida State University, Florida.

[B85] Tannenbaum R. J. Wylie E. C. (2013). “Mapping the TOEIC^®^ and TOEIC Bridge^TM^ test scores to the Common European Framework of Reference,” in *The Research Foundation for the TOEIC tests: A Compendium of Studies*, Vol. II, ed. Powers D. E. (Princeton, NJ: Educational Testing Service), 6.1–6.10.

[B86] Tseng W.-T. (2011). *Modeling Vocabulary Knowledge: A Mixed Model Approach*. Paper presented at 2011 Language Testing Research Colloquium. Ann Arbor: University of Michigan.

[B87] Tseng W.-T. Schmitt N. (2008). Toward a model of motivated vocabulary learning: a structural equation modeling approach. *Lang. Learn.* 58 357–400. 10.1111/j.1467-9922.2008.00444x

[B88] Twilley L. C. Dixon P. Taylor D. Clark K. (1994). University of Alberta norms of relative meaning frequency for 566 homographs. *Mem. Cogn.* 22 111–126. 10.3758/BF03202766 8035680

[B89] Vafaee P. (2016). *The Relative Significance of Syntactic Knowledge and Vocabulary Knowledge in Second Language Listening Comprehension.* PhD dissertation, University of Maryland, Maryland, 10.13016/M2B485.

[B90] Vafaee P. Kachinske I. (2019). The inadequate use of confirmatory factor analysis in second language acquisition validation studies. *Stud. Appl. Linguist. TESOL* 19 1–18. 10.7916/salt.v19i2.4184

[B91] Vafaee P. Suzuki S. (2020). The relative significance of syntactic knowledge and vocabulary knowledge in second language listening ability. *Stud. Sec. Lang. Acquis.* 10.1017/S0272263119000676

[B92] Vermeer A. (2001). Breadth and depth of vocabulary in relation to L1/L2 acquisition and frequency of input. *Appl. Psycholinguist.* 22 217–234. 10.1017/S0142716401002041

[B93] Vilkaitė-Lozdienė L. Schmitt N. (2020). “Frequency as a guide for vocabulary usefulness: high-, mid-, and low-frequency words,” in *The Routledge Handbook of Vocabulary Studies*, ed. Webb S. (London: Routledge), 81–96.

[B94] Webb S. (2005). Receptive and productive vocabulary learning: the effects of reading and writing on word knowledge. *Stud. Sec. Lang. Acquis.* 27 33–52. 10.1017/S0272263105050023

[B95] Webb S. (2013). “Depth of vocabulary knowledge,” in *Encyclopedia of Applied Linguistics*, ed. Chapelle C. (Oxford: Wiley-Blackwell), 1656–1663. 10.1002/9781405198431.wbeal1325

[B96] Webb S. Nation P. (2017). *How Vocabulary is Learned.* Oxford: Oxford University Press.

[B97] Wesche M. Paribakht T. S. (1993). Assessing second language vocabulary knowledge: depth versus breadth. *Can. Modern Lang. Rev.* 53 13–40.

[B98] Winke P. (2014). Testing hypotheses about language learning using structural equation modeling. *Ann. Rev. Appl. Linguist.* 34 102–122. 10.1017/S0267190514000075

[B99] Yamashita J. Shiotsu T. (2017). Comprehension and knowledge components that predict L2 Reading: a latent-trait approach. *Appl. Linguist.* 38 43–67. 10.1093/applin/amu079

[B100] Yan X. Cheng L. Ginther A. (2019). Factor analysis for fairness: examining the impact of task type and examinee L1 background on scores of an ITA speaking test. *Lang. Test.* 36 207–234. 10.1177/0265532218775764

[B101] Yanagisawa A. Webb S. (2020). “Measuring depth of vocabulary knowledge,” in *The Routledge Handbook of Vocabulary Studies*, ed. Webb S. (London, UK: Routledge), 371–386.

[B102] yWorks GmbH (2000–2019). *yEd Graph Editor (Version 3.19.1.1) [Computer software].* Germany: yWorks.

[B103] Zhang D. (2012). Vocabulary and grammar knowledge in second language reading comprehension: a structural equation modeling study. *Modern Lang. J.* 96 558–575. 10.2307/23361716

[B104] Zhang L. Goh C. C. M. Kunnan A. J. (2014). Analysis of test takers’ metacognitive and cognitive strategy use and EFL reading test performance: a multi-sample SEM approach. *Lang. Assess. Q.* 11 76–102. 10.1080/15434303.2013.853770

