# Supplementary material for: Structural Equation Modeling of Vocabulary Size and Depth Using Conventional and Bayesian Methods
Source: Front Psychol. 2020 Apr 21;11:618. doi: 10.3389/fpsyg.2020.00618 (PMC7187790; doi:10.3389/fpsyg.2020.00618)
Supplement: Supplementary file 1 [file Data_Sheet_1.PDF]

Supplementary materials for "Structural equation modeling of vocabulary size and depth using conventional and Bayesian methods"

Appendix A: Five vocabulary tests used in the current study (Mochizuki et al., 2014)

これは単語のさまざまな知識を問うテストです。全部で 130 題あります。1 題を解答するのに 30 秒を目安に，全体で 80 分間で解答してください。

[This test assesses various aspects of your vocabulary knowledge. There are 130 items. Use approximately 30 seconds per item and answer all items in a total of 80 minutes.]

[For article readers: Answers are highlighted in yellow.]

I　例題に従って，問題の語から一番強く連想する語を (1)～ (4)の中から 1 つ選び，数字をマークしなさい。1～30.

[*Word Association Test* (30 items; No. 1 to 30): Select the English word from options 1 through 4 that is most strongly associated with the given word.]

例 (Example) 1. sky

(1) blue　(2) yellow　(3) white　(4) black

正解は (1)，理由は blue sky という表現から

[The answer is 1 because the word *sky* is associated the most strongly with the word *blue* as in the expression *blue sky*.]

例 (Example) 2. run

(1) jog　(2) skip　(3) sleep　(4) throw

正解は (1)，理由は run は jog と意味が近いから

[The answer is 1 because the words *run* and *jogs* have similar meanings.]

1. attack

(1) action	(2) defend	(3) guard	(4) shout

2. become

(1) begin	(2) grow	(3) season	(4) start

3. communicate

(1) chat	(2) convey	(3) express	(4) meet

4. feel

(1) cry	(2) keep	(3) match	(4) touch

5. kind

(1) careful	(2) familiar	(3) hard	(4) gentle

6. real

(1) fantasy	(2) fiction	(3) image	(4) television

7. remember

(1) leave	(2) notice	(3) recall	(4) think

8. save

(1) destroy	(2) help	(3) lose	(4) receive

9. social

(1) community	(2) company	(3) country	(4) school

10. style

(1) figure	(2) thick	(3) thin	(4) weight

11. success

(1) company	(2) document	(3) lot	(4) promotion

12. surprised

(1) astonished	(2) confused	(3) excited	(4) taken

13. training

(1) walk	(2) sport	(3) dash	(4) running

14. tree

(1) chair	(2) desk	(3) park	(4) plant

15. wide

(1) broad	(2) full	(3) high	(4) spacious

16. attack

(1) ball	(2) heart	(3) night	(4) plane

17. become

(1) cool	(2) interested	(3) old	(4) sick

18. communicate

(1) classmate	(2) letter	(3) school	(4) telephone

19. feel

(1) angry	(2) glad	(3) sick	(4) well

20. kind

(1) father	(2) gentleman	(3) men	(4) person

21. real

(1) money	(2) number	(3) telephone	(4) world

22. remember

(1) dinner	(2) promise	(3) school	(4) word

23. save

(1) animal	(2) life	(3) load	(4) sports

24. social

(1) action	(2) enemy	(3) order	(4) problem

25. style

(1) dance	(2) change	(3) life	(4) model

26. success

(1) great (2) huge (3) small (4) wonderful

27. surprised

(1) birthday (2) boy (3) friend (4) news

28. training

(1) arm (2) body (3) diet (4) dog

29. tree

(1) branch (2) house (3) orange (4) root

30. wide

(1) face (2) picture (3) range (4) trade

II　問題の語を表す意味を (1)～ (5)の中から 2 つ選び，数字をマークしなさい。マークは同じ行にマークしなさい。31～50.

[*Polysemy Test 1* (20 items; No. 31 to 50): Select the two Japanese meanings from options 1 through 5 that correspond to the English word provided.

31. court

(1) 塗装 (2) 任命 (3) 王宮 (4) 狩猟 (5) 法廷

32. fire

(1) 命令する (2) 解雇する (3) 目撃する (4) 発射する (5) 無視する

33. article

(1) 記事 (2) 粒 (3) 芸術 (4) 品物 (5) 捜索

34. fair

(1) 安全な (2) 適正な (3) 運命的な (4) 多様な (5) 公平な

35. introduce

(1) 導入する (2) 演奏する (3) 紹介する (4) 反応する (5) 解説する

36. order

(1) 機能 (2) 順序 (3) 依頼 (4) 試合 (5) 命令

37. trial

(1) 裁判 (2) 試み (3) 改良 (4) 契約 (5) 解散

38. present

(1) 明らかな (2) 楽しい (3) 表面の (4) 出席している (5) 現在の

39. capital

(1) 軍隊 (2) 紛争 (3) 資本 (4) 首都 (5) 高い塔

40. thick

(1) 裕福な (2) 厚い (3) 込み合った (4) 古びた (5) 真実の

41. change

(1) 小銭 (2) 動力 (3) 政策 (4) 電圧 (5) 変化

42. succeed

(1) 運搬する (2) 後を継ぐ (3) 成功する (4) 整頓する (5) 約束する

43. rest

(1) 休息 (2) 残り (3) 食事 (4) 信頼 (5) 予約

44. case

(1) 基準 (2) 商品 (3) 事件 (4) 場合 (5) 封鎖

45. object

(1) 価値 (2) 科目 (3) 分類 (4) 目的 (5) 物

46. stock

(1) 株 (2) 推薦 (3) 在庫品 (4) 靴下 (5) 補助

47. issue

(1) 出版物 (2) 調和 (3) 訂正 (4) 問題 (5) 組織

48. stick

(1) 支える (2) 曲げる (3) 貼り付ける (4) 削る (5) 突き刺す

49. feature

(1) 顔立ち (2) 銅像 (3) 参加 (4) 広告 (5) 特徴

50. practice

(1) 連帯 (2) 慣例 (3) 改良 (4) 練習 (5) 忍耐

III　問題の日本語に相当する英単語を (1)～ (4)の中から 1 つ選び，数字をマークしなさい。51～70.

[*Polysemy Test 2* (20 items; No. 51 to 70; 17 items were analyzed in the current study; 3 items—58, 69, and 70—were removed from the analysis because they were found to assess knowledge of the first definition, which overlapped the concept of vocabulary size): Select the English word from options 1 through 4 that corresponds to the Japanese meaning provided.

51. 弾丸

(1) battery (2) congress (3) ally (4) bullet

52. 仲間

(1) criminal (2) crisis (3) expert (4) fellow

53. …を詰める

(1) ban (2) attempt (3) stuff (4) grasp

54. (思想，信条）を断念する・捨てる

(1) abandon (2) confuse (3) deserve (4) absorb

55. (人の注意）を引く

(1) arrest (2) check (3) search (4) cause

56. うわさ，世評

(1) reflection (2) spectrum (3) demonstration (4) reputation

57. 侵害

(1) destination (2) invasion (3) assumption (4) nightmare

58. 信仰，信念 (Not included in the analysis)

(1) soul (2) condition (3) faith (4) decision

59. 比較

(1) competition (2) comparison (3) diversity (4) triumph

60. 認知，知覚

(1) impression (2) perception (3) action (4) emotion

61. (人に性的に）惹かれる

(1) rub (2) adjust (3) fancy (4) devote

62. 過労，負担・重圧

(1) sacrifice (2) discipline (3) ruin (4) strain

63. (人）を甘やかす

(1) react (2) spoil (3) interfere (4) rid

64. (人）から…を取り除く

(1) recover (2) relieve (3) enable (4) bury

65. (…に）屈する・従う

(1) bow (2) bowl (3) burst (4) bar

66. 伴奏する

(1) dioxide (2) accompany (3) compose (4) shrug

67. へり，縁，端

(1) patch (2) border (3) poverty (4) band

68 . (数，量が）急増する

(1) indicate (2) explore (3) vary (4) explode

69. 少量 (Not included in the analysis)

(1) grain (2) lap (3) ton (4) salt

70. …に (フィルム・カートリッジ）を装てんする (Not included in the analysis)

(1) command (2) load (3) spread (4) climb

IV 「英語として」問題の語に結びついて意味の通る語を，(1)〜 (4)の中から 2 つ選び，数字をマークしなさい。マークは同じ行にマークしなさい。ただし，選択肢の語は (　　)のある位置に来るものとします。71〜90.

[*Collocation Test* (20 items; No. 71 to 90): Select two words from options 1 through 4 that make a coherent meaning when each is combined with the word provided in English. Selected words appear in the place of (　　) displayed before or after the word provided.]

例. short (　　)

(1) salt (2) shop (3) time (4) supply

答. (3) (short time 「短い時間」) (4) (short supply 「不足している供給」)

[The answers are 3 and 4 because the expressions *short time* (mijikai jikan) and *short supply* (fusoku shiteiru kyokyu) co-occur frequently.]

71. heavy (　　)

(1) door (2) mathematics (3) meal (4) sunshine

72. rough (　　)

(1) road (2) fun (3) lunch (4) area

73. late (　　)

(1) aim (2) night (3) computer (4) lunch

74. loud (　　)

(1) suit (2) coffee (3) silence (4) noise

75. wild (　　)

(1) animal (2) guess (3) pencil (4) pocket

76. green (　　)

(1) air (2) kick (3) fingers (4) leaves

77. rich (   )

(1) smell (2) clock (3) people (4) laundry

78. fast (   )

(1) tree (2) food (3) sugar (4) color

79. long (   )

(1) face (2) money (3) time (4) tea

80. tall (   )

(1) winter (2) man (3) order (4) paper

81. black (   )

(1) hair (2) cycling (3) flight (4) look

82. bright (   )

(1) gas (2) girl (3) star (4) storm

83. easy (   )

(1) day (2) eye (3) queen (4) question

84. soft (   )

(1) distance (2) storm (3) job (4) bed

85. flat (   )

(1) beer (2) tower (3) rain (4) area

86. clean (   )

(1) record (2) regain (3) refuse (4) room

87. strong (   )

(1) coffee (2) coward (3) winds (4) date

88. hot (   )

(1) day (2) issue (3) snow (4) darkness

89. sharp (   )

(1) sponge (2) circle (3) increase (4) knife

90. poor (   )

(1) honesty (2) people (3) judgment (4) eternity

V　問題の日本語の意味を表す単語を (1)～ (4)の中から 1 つ選び，数字をマークしなさい。 91~130.

[*Size Test* (40 items; No. 91 to 130): Select the English word from options 1 through 4 that best corresponds to the Japanese meaning provided.]

91. 話に出す, 言及する

(1) manipulate (2) mention (3) minister (4) moderate

92. 返事をする, 返事

(1) receive (2) relay (3) repeat (4) reply

93. 十字架, 横切る

(1) class (2) close (3) course (4) cross

94. 存在, 生き物

(1) bean (2) beast (3) being (4) brain

95. 最も少ない

(1) least (2) less (3) little (4) lit

96. 語句

(1) passage (2) phrase (3) praise (4) pronoun

97. 哲学

(1) philology (2) philosophy (3) physiology (4) psychology

98. 宣言する, 申告する

(1) decade (2) declare (3) demand (4) deny

99. 狙う, ねらい

(1) aim (2) assign (3) attend (4) avoid

100. 取り囲む

(1) signal (2) solve (3) spread (4) surround

101. 不具になった, 身体障害のある

(1) desperate (2) delighted (3) disabled (4) dull

102. 重要な, 意味のある

(1) sacred (2) severe (3) significant (4) solid

103. 批評, 批判

(1) canal (2) celebration (3) civilization (4) criticism

104. 訓練, しつけ

(1) declaration (2) discipline (3) drawer (4) duke

105. 詳細な

(1) decent (2) delicate (3) delighted (4) detailed

106. 論争

(1) chase (2) coalition (3) controversy (4) conversation

107. 観察者, 傍聴者

(1) objector (2) observer (3) opportunity (4) opposite

108. 孤立

(1) incentive (2) indent (3) infant (4) isolation

109. 忠誠, 忠誠心

(1) lateral (2) lawful (3) liberal (4) loyalty

110. 部門, 分野

(1) sector (2) shape (3) statement (4) substance

111. 群れ

(1) herd (2) hangar (3) hummer (4) hunger

112. 巨大な, 多大な

(1) illegal (2) immense (3) infamous (4) irregular

113. 可能性, 見込み

(1) likelihood (2) lethal (3) lexicon (4) lunatic

114. 住まい

(1) deploy (2) destine (3) diva (4) dwelling

115. 苦々しく

(1) beadily (2) belatedly (3) bitterly (4) busily

116. 信条，信念

(1) creed (2) contention (3) coincidence (4) contraction

117. 通行料，被害，犠牲者

(1) tomb (2) tract (3) trait (4) toll

118. 倫理学，道義，道徳

(1) excavation (2) ethics (3) enclosure (4) entitlement

119. 成長する，栄える

(1) tumble (2) tread (3) tilt (4) thrive

120. 尊敬すべき，立派な

(1) harmful (2) hybrid (3) honorable (4) humble

121. 予言者

(1) potency (2) prophet (3) preacher (4) protester

122. 除去，削除

(1) endorsement (2) elimination (3) endowment (4) enhancement

123. 気前のよさ，寛大

(1) granny (2) granite (3) generosity (4) geology

124. 噴火する，噴出する

(1) elicit (2) erupt (3) erode (4) evacuate

125. 皮肉な，矛盾する

(1) indicative (2) intriguing (3) ironic (4) intrinsic

126. 恐怖，おびえ

(1) friction (2) fragrance (3) fright (4) fertilizer

127. 青春期，思春期

(1) anthem (2) assassin (3) atrocity (4) adolescence

128. 授ける，贈与する

(1) bestow (2) beckon (3) banish (4) brighten

129. 怒らせる，挑発的な

(1) perennial (2) precarious (3) prolific (4) provocative

130. 仮定上の，仮説の

(1) horrific (2) humane (3) hypothetical (4) hysterical

frontiers

Appendix B: Frequency of words in the tests

The tests were developed on the basis of JACET8000 (JACET Basic Word Revision Committee, 2003) and analyzed using the updated version of JACET8000 (JACET Basic Word Revision Committee, 2016). The level in the table shows word frequency in JACET8000 (JACET Basic Word Revision Committee, 2016). When word frequency was different from our intention at the development stage, we also checked JACET8000 (JACET Basic Word Revision Committee, 2003) and reported the results in parentheses. For example, Item 11 had the word *promotion* among its options. Its word frequency was reported to be 4 (3). The results indicate that its word frequency was 4 in JACET8000 (JACET Basic Word Revision Committee, 2016), which shows that the word is at the 4,000-lemma level. The results also indicate that its word frequency was 3 in JACET8000 (JACET Basic Word Revision Committee, 2003), which shows that the word is at the 3,000-lemma level.

Item No.	Word	L	Option1	L	Option2	L	Option3	L	Option4	L
Word Association										
Example 1	sky	1	blue	1	yellow	1	white	1	black	1
Example. 2	run	1	jog	4	skip	3	sleep	1	throw	1
**1**	attack	2	action	1	defend	3	guard	2	shout	1
**2**	become	1	begin	1	grow	1	season	1	start	1
**3**	communicate	2	chat	4	convey	4	express	1	meet	1
**4**	feel	1	cry	1	keep	1	match	1	touch	1

**5**	kind	1	careful	1	familiar	2	hard	1	gentle	2
**6**	real	1	fantasy	6	fiction	2	image	2	television	3
**7**	remember	1	leave	1	notice	1	recall	3	think	1
**8**	save	1	destroy	2	help	1	lose	1	receive	1
**9**	social	1	community	2	company	1	country	1	school	1
**10**	style	1	figure	1	thick	2	thin	2	weight	2
**11**	success	2	company	1	document	2	lot	1	promotion	4 (3)
**12**	surprised	1	astonished	NA (aston-ish 7)	confused	2	excited	1	taken	1
**13**	training	1	walk	1	sport	1	dash	5	running	1
**14**	tree	1	chair	1	desk	1	park	1	plant	1
**15**	wide	1	broad	3	full	1	high	1	spacious	5
**16**	attack	2	ball	1	heart	1	night	1	plane	1
**17**	become	1	cool	1	interested	1	old	1	sick	1

**18**	communicate	2	classmate	2	letter	1	school	1	telephone	1
**19**	feel	1	angry	1	glad	1	sick	1	well	1
**20**	kind	1	father	1	gentleman	4	men	1	person	1
**21**	real	1	money	1	number	1	telephone	1	world	1
**22**	remember	1	dinner	1	promise	2	school	1	word	1
**23**	save	1	animal	1	life	1	load	3	sports	1
**24**	social	1	action	1	enemy	2	order	1	problem	1
**25**	style	1	dance	1	change	1	life	1	model	2
**26**	success	2	great	1	huge	2	small	1	wonderful	1
**27**	surprised	1	birthday	1	boy	1	friend	1	news	1
**28**	training	1	arm	1	body	1	diet	2	dog	1
**29**	tree	1	branch	2	house	1	orange	1	root	2
**30**	wide	1	face	1	picture	1	range	2	trade	2

Polysemy 1

**31**	court	2
**32**	fire	1
**33**	article	2
**34**	fair	1
**35**	introduce	1
**36**	order	1
**37**	trial	2
**38**	present	1
**39**	capital	2
**40**	thick	2
**41**	change	2
**42**	succeed	1
**43**	rest	1
**44**	case	1
**45**	object	2

**46**	stock	3 (2)
**47**	issue	2
**48**	stick	2
**49**	feature	1
**50**	practice	1

Polysemy 2

**51**	battery	3	congress	2	ally	4	bullet	4 (3)
**52**	criminal	3	crisis	3	expert	2	fellow	3
**53**	ban	2	attempt	1	stuff	3	grasp	5
**54**	abandon	3	confuse	2	deserve	3	absorb	2
**55**	arrest	2	check	1	search	2	cause	1
**56**	reflection	5	spectrum	5	demonstration	3	reputation	3
**57**	destination	3	invasion	4 (3)	assumption	3	nightmare	5
~~**58**~~	~~soul~~	~~4~~	~~condition~~	~~2~~	~~faith~~	~~4~~	~~decision~~	~~2~~

**59**			competition	2	comparison	3	diversity	3	triumph	6
**60**			impression	2	perception	3	action	1	emotion	2
**61**			rub	4	adjust	3	fancy	4 (3)	devote	3
**62**			sacrifice	5	discipline	3	ruin	3	strain	4 (3)
**63**			react	3	spoil	3	interfere	4	rid	2
**64**			recover	2	relieve	3	enable	3	bury	2
**65**			bow	4 (2)	bowl	2	burst	3	bar	2
**66**			dioxide	3	accompany	3	compose	3	shrug	6
**67**			patch	5	border	2	poverty	2	band	1
**68**			indicate	3	explore	2	vary	2	explode	3
~~**69**~~			~~grain~~	~~3~~	~~lap~~	~~4~~	~~ton~~	~~2~~	~~salt~~	~~2~~
~~**70**~~			~~command~~	~~2~~	~~load~~	~~3~~	~~spread~~	~~1~~	~~climb~~	~~1~~
Collocation										
Example	short	1	salt	2	shop	2	time	1	supply	2
**71**	heavy	1	door	1	mathematics	2	meal	1	sunshine	4

**72**	rough	2	road	1	fun	1	lunch	1	area	1
**73**	late	1	aim	2	night	1	computer	1	lunch	1
**74**	loud	1	suit	2	coffee	1	silence	2	noise	2
**75**	wild	1	animal	1	guess	1	pencil	2	pocket	2
**76**	green	1	air	2	kick	2	fingers	2	leaves	1
**77**	rich	1	smell	1	clock	1	people	1	laundry	4
**78**	fast	1	tree	1	food	1	sugar	2	color	1
**79**	long	1	face	1	money	1	time	1	tea	1
**80**	tall	1	winter	1	man	1	order	1	paper	1
**81**	black	1	hair	1	cycling	1	flight	1	look	1
**82**	bright	1	gas	2	girl	1	star	1	storm	2
**83**	easy	1	day	1	eye	1	queen	3	question	1
**84**	soft	1	distance	2	storm	2	job	1	bed	1
**85**	flat	2	beer	4 (3)	tower	1	rain	1	area	1

**86**	clean	1	record	1	regain	5	refuse	2	room	1
**87**	strong	1	coffee	1	coward	NA	winds	1	date	1
**88**	hot	1	day	1	issue	2	snow	1	darkness	4
**89**	sharp	2	sponge	8	circle	2	increase	1	knife	2
**90**	poor	1	honesty	4	people	1	judgment	3	eternity	6
Size										
**91**			manipulate	6	mention	2 (1)	minister	3	moderate	4
**92**			receive	1	relay	5	repeat	2	reply	2 (1)
**93**			class	1	close	1	course	1	cross	2 (1)
**94**			bean	2	beast	6	being	2 (1)	brain	2
**95**			least	2 (1)	less	1	little	1	lit	1
**96**			passage	3	phrase	3 (2)	praise	2	pronoun	NA (8)
**97**			philology	NA	philosophy	3 (2)	physiology	6	psychology	3
**98**			decade	2	declare	3 (2)	demand	2	deny	3

**99**	aim	1 (2)	assign	3	attend	2	avoid	2
**100**	signal	2	solve	1	spread	1	surround	2 (2)
**101**	desperate	5	delighted	4	disabled	4 (3)	dull	4
**102**	sacred	4	severe	2	significant	3 (3)	solid	3
**103**	canal	5	celebration	3	civilization	4	criticism	3 (3)
**104**	declaration	5	Discipline	NA (3)	drawer	4	duke	6
**105**	decent	5	delicate	3	delighted	4	detailed	2 (3)
**106**	chase	3	coalition	5	controversy	5 (4)	conversation	2
**107**	objector	NA	observer	5 (4)	opportunity	2	opposite	2
**108**	incentive	4	indent	NA	infant	5	isolation	5 (4)
**109**	lateral	7	lawful	NA (7)	liberal	5	loyalty	5 (4)
**110**	sector	4 (4)	shape	2	statement	2	substance	3
**111**	herd	6 (5)	hangar	NA	hummer	NA	hunger	3
**112**	illegal	2	immense	5 (5)	infamous	8	irregular	6

**113**	likelihood	5 (5)	lethal	7	lexicon	8	lunatic	NA (8)
**114**	deploy	7	destine	7	diva	NA	dwelling	6 (5)
**115**	beadily	NA	belatedly	NA	bitterly	7 (5)	busily	NA
**116**	creed	8 (6)	contention	7	coincidence	5	contraction	8
**117**	tomb	5	tract	7	trait	5	toll	5 (6)
**118**	excavation	5	ethics	NA (6)	enclosure	6	entitlement	8
**119**	tumble	8	tread	NA (6)	tilt	5	thrive	3
**120**	harmful	3	hybrid	4	honorable	NA (6)	humble	7
**121**	potency	NA (7)	prophet	8 (7)	preacher	8	protester	7
**122**	endorsement	7	elimination	6 (7)	endowment	7	enhancement	6
**123**	granny	1	granite	7	generosity	7 (7)	geology	5
**124**	elicit	NA (7)	erupt	6 (7)	erode	5	evacuate	6
**125**	indicative	8	intriguing	NA (7)	ironic	7 (7)	intrinsic	7
**126**	friction	7	fragrance	8	fright	8 (8)	fertilizer	4

**127**	anthem	8	assassin	NA (8)	atrocity	NA (8)	adolescence	7 (8)
**128**	bestow	NA (8)	beckon	NA (8)	banish	NA (8)	brighten	NA (8)
**129**	perennial	8	precarious	NA (8)	prolific	8	provocative	7 (8)
**130**	horrific	7	humane	8	hypothetical	8 (8)	hysterical	8

Note. L = JACET8000 frequency level (examined using the New Word Level Checker: https://nwlc.pythonanywhere.com/). 1 = 1000-lemma level. ( ) = Frequency level using JACET8000 (JACET Basic Word Revision Committee, 2003, examined using the wordlist found at http://language.sakura.ne.jp/s/voc.html).

Appendix C: Analysis of collocation test items

We examined the 20 items using the Corpus of Contemporary American English (COCA) and also the British National Corpus (BNC), when needed; the results are shown below. There were four items whose correct word combinations had MI scores of less than 1.0 using the COCA and the BNC (i.e., Item Nos. 78, 81, 83, and 84). These items were not considered highly problematic because two experienced Japanese teachers of English and an English native speaker confirmed that correct options co-occurred frequently and distractors least collocated with the stimulus word, and also because there were no other options that could be correct. However, future revisions could be made to improve the quality of these items.

Additionally, there was one item (i.e., Item No. 74) whose incorrect word combination (*loud silence*) had a high MI score of 3.40, which was as high as one of the correct word combinations (3.56; *loud suit*), using the COCA. Given that this item could have a different correct option, we reanalyzed the data by excluding this potentially problematic item. Results showed that the outcome was almost the same (i.e., both $\alpha = .77$; r differences = .00 to .01). Collocation scores with 20 items were very strongly correlated with those with 19 items ($r = .991$). Thus, we reported the analysis using 20 collocation items.

	Item No.	Word	Option 1	Option 2	Option 3	Option 4
	Example	short	salt	shop	time	supply
Frequency			3	0	6665	1300
MI			NA	NA	4.68	7.51
	71	heavy	door	mathematics	meal	sunshine
Frequency			244	2	38	0
MI			4.04	NA	4.19	NA
	72	rough	road	fun	lunch	area
Frequency			162	1	0	32
MI			5.30	NA	NA	2.44
	73	late	aim	night	computer	lunch

Frequency			0	2607	1	169
MI			NA	5.03	NA	4.12
	74	loud	suit	coffee	silence	noise
Frequency			5	1	15	431
MI			3.56 (4.84)	NA	3.40 (2.96)	8.47 (8.65)
	75	wild	animal	guess	pencil	pocket
Frequency			759	284	0	2
MI			7.81	4.96	NA	NA
	76	green	air	kick	fingers	leaves
Frequency			7	2	19	623
MI			NA	NA	1.60	6.21
	77	rich	smell	clock	people	laundry
Frequency			36	0	2685	0
MI			3.38	NA	4.14	NA
	78	fast	tree	food	sugar	color
Frequency			0	3454	1	9
MI				7.29		-0.41 (NA)
	79	long	face	Money	time	tea
Frequency			350	9	60390	2
MI			0.97 (1.14)	NA	5.92	NA

	80	tall	winter	Man	order	paper
Frequency			0	1016	605	5
MI			NA	5.10	6.09	NA
	81	black	hair	cycling	flight	look
Frequency			4089	1	19	26
MI			6.55	NA	NA	-3.22 (0.25)
	82	bright	gas	girl	star	storm
Frequency			7	66	431	0
MI			0.65	2.72	6.30	NA
	83	easy	day	eye	queen	question
Frequency			121	9	0	207
MI			-0.02 (0.78)	NA	NA	2.07
	84	soft	distance	storm	job	bed
Frequency			2	0	17	115
MI			NA	NA	0.18 (NA)	4.39
	85	flat	beer	tower	rain	area
Frequency			18	0	1	83
MI			3.35	NA	NA	3.28
	86	clean	record	regain	refuse	room
Frequency			147	0	0	211

MI			3.74	NA	NA	2.99
	87	strong	coffee	coward	winds	date
Frequency			176	0	675	0
MI			4.04	NA	7.96	NA
	88	hot	day	issue	snow	darkness
Frequency			784	176	3	7
MI			3.14	2.85	NA	NA
	89	sharp	sponge	circle	increase	knife
Frequency			0	3	329	708
MI			NA	NA	6.37	9.45
	90	poor	honesty	people	judgment	eternity
Frequency			0	4914	520	0
MI			NA	4.49	7.05	NA

Note. Frequency = COCA's frequency. MI = COCA's mutual information (MI) score. ( ) = BNC MI score. NA = No MI scores were provided.

Appendix D: Mplus code for structural equation modeling (Models 1 and 2)

```
Title: Model 1 single factor
Data:
   File="H24_N255_k127_size_depth.dat";
Variable:
   NAMES = id_number Associ Poly1 Poly2 Colloca Size Size123 Size456 Size78;
   USEVARIABLES = Associ Poly1 Poly2 Colloca Size123 Size456 Size78;
Define:
   standardize Associ Poly1 Poly2 Colloca Size123 Size456 Size78;
Analysis:
   ESTIMATOR=MLR;
Model:
   SizeDepth BY Size123 Size456 Size78 Associ Poly1 Poly2 Colloca;
Output:
   STAND(STDYX) sampstat stand tech4 stdyx tech8;


Title: Model 2 two correlated
Data:
   File="H24_N255_k127_size_depth.dat";
Variable:
   NAMES = id_number Associ Poly1 Poly2 Colloca Size Size123 Size456 Size78;
   USEVARIABLES = Associ Poly1 Poly2 Colloca Size123 Size456 Size78;
Define:
   standardize Associ Poly1 Poly2 Colloca Size123 Size456 Size78;
Analysis:
   ESTIMATOR=MLR;
```

```
Model:
    Size BY Size123 Size456 Size78;
    Depth BY Associ Poly1 Poly2 Colloca;
    Size WITH Depth;
Output:
    STAND(STDYX) sampstat stand tech4 stdyx tech8;
```

Appendix E: Mplus code for Bayesian structural equation modeling with noninformative priors (Models 1a and 2a)

```
Title: Model 1a single factor
Data:
    File="H24_N255_k127_size_depth.dat";
Variable:
    NAMES = id_number Associ Poly1 Poly2 Colloca Size Size123 Size456 Size78;
    USEVARIABLES = Associ Poly1 Poly2 Colloca Size123 Size456 Size78;
Define:
    standardize Associ Poly1 Poly2 Colloca Size123 Size456 Size78;
Analysis:
    TYPE=GENERAL;
    ESTIMATOR=BAYES;
    POINT=MEDIAN;
    CHAIN=3;
    PROCESSORS=2;
    BITERATIONS=(10000);
Model:
```

```
   SizeDepth BY Size123 Size456 Size78 Associ Poly1 Poly2 Colloca;
Output:
  STAND(STDYX) sampstat stand tech4 stdyx tech8;
Plot: type=plot2;


Title: Model 2a two correlated
Data:
  File="H24_N255_k127_size_depth.dat";
Variable:
  NAMES = id_number Associ Poly1 Poly2 Colloca Size Size123 Size456 Size78;
  USEVARIABLES = Associ Poly1 Poly2 Colloca Size123 Size456 Size78;
Define:
  standardize Associ Poly1 Poly2 Colloca Size123 Size456 Size78;
Analysis:
  TYPE=GENERAL;
  ESTIMATOR=BAYES;
  POINT=MEDIAN;
  CHAIN=3;
  PROCESSORS=2;
  BITERATIONS=(10000);
Model:
   Size BY Size123 Size456 Size78;
   Depth BY Associ Poly1 Poly2 Colloca;
   Size WITH Depth;
Output:
  STAND(STDYX) sampstat stand tech4 stdyx tech8;
```

```
Plot: type=plot2;
```

Appendix F: Mplus code for Bayesian structural equation modeling with informative, small-variance priors for cross-loadings (Model 2b)

```
Title: Model 2b
Data:
   File="H24_N255_k127_size_depth.dat";
Variable:
   NAMES = id_number Associ Poly1 Poly2 Colloca Size Size123 Size456 Size78;
   USEVARIABLES = Associ Poly1 Poly2 Colloca Size123 Size456 Size78;
Define:
   standardize Associ Poly1 Poly2 Colloca Size123 Size456 Size78;
Analysis:
   TYPE=GENERAL;
   ESTIMATOR=BAYES;
   POINT=MEDIAN;
   CHAIN=3;
   PROCESSORS=2;
   BITERATIONS=(10000);
   fbiterations=100000;!10000 produced error in Kolmogorov!
Model:
    Size BY Size123 Size456 Size78;
    Depth BY Associ Poly1 Poly2 Colloca;
    Size WITH Depth;
    Size BY Associ-Colloca*0(cross1-cross4);!cross loadings!
```

```
  Depth BY Size123-Size78*0(cross5-cross7);!cross loadings!
Model priors:
 cross1-cross4~N(0,0.01);
 cross5-cross7~N(0,0.01);
Output:
   STAND(STDYX) sampstat stand tech4 stdyx tech8;
Plot: type=plot2;
```

Appendix G: Mplus code for Bayesian structural equation modeling with informative, small-variance priors for residual variances (Model 1c)

```
Title: Model 1c
Data:
   File="H24_N255_k127_size_depth.dat";
Variable:
   NAMES = id_number Associ Poly1 Poly2 Colloca Size Size123 Size456 Size78;
   USEVARIABLES = Associ Poly1 Poly2 Colloca Size123 Size456 Size78;
Define:
   standardize Associ Poly1 Poly2 Colloca Size123 Size456 Size78;
Analysis:
   TYPE=GENERAL;
   ESTIMATOR=BAYES;
   POINT=MEDIAN;
   CHAIN=3;
   PROCESSORS=2;
   BITERATIONS=(10000);
```

```
    fbiterations=100000;
Model:
   SizeDepth BY Size123 Size456 Size78 Associ Poly1 Poly2 Colloca;
   Associ-Colloca(residual1-residual4);!uncorrelated residuals!
   Size123-Size78(residual5-residual7);!uncorrelated residuals!
   Associ Poly1 Poly2 Colloca Size123 Size456 Size78 with
   Associ Poly1 Poly2 Colloca Size123 Size456 Size78(residual8-residual28);!uncorrelated
Model priors:
 residual1-residual4~IW(0,14);!degrees of freedom =14!
 residual5-residual7~IW(0,14);!degrees of freedom =14!
 residual8-residual28~IW(0,14);!degrees of freedom =14!
Output:
  STAND(STDYX) sampstat stand tech4 stdyx tech8;;
Plot: type=plot2;
```

Appendix H: Mplus code for Bayesian structural equation modeling with informative, small-variance priors for cross-loadings and residual variances (Model 2c)

```
Title: Model 2c
Data:
  File="H24_N255_k127_size_depth.dat";
Variable:
  NAMES = id_number Associ Poly1 Poly2 Colloca Size Size123 Size456 Size78;
  USEVARIABLES = Associ Poly1 Poly2 Colloca Size123 Size456 Size78;
Define:
```

```
    standardize Associ Poly1 Poly2 Colloca Size123 Size456 Size78;
Analysis:
    TYPE=GENERAL;
    ESTIMATOR=BAYES;
    POINT=MEDIAN;
    CHAIN=3;
    PROCESSORS=2;
    BITERATIONS=(10000);
    fbiterations=100000;!10000 produced error in Kolmogorov!
Model:
     Size BY Size123 Size456 Size78;
     Depth BY Associ Poly1 Poly2 Colloca;
     Size WITH Depth;
     Size BY Associ-Colloca*0(cross1-cross4);!cross loadings!
     Depth BY Size123-Size78*0(cross5-cross7);!cross loadings!
     Associ-Colloca(residual1-residual4);!uncorrelated residuals!
     Size123-Size78(residual5-residual7);!uncorrelated residuals!
     Associ Poly1 Poly2 Colloca Size123 Size456 Size78 with
     Associ Poly1 Poly2 Colloca Size123 Size456 Size78(residual8-residual28);!correlated residuals!
Model priors:
cross1-cross4~N(0,0.01);
cross5-cross7~N(0,0.01);
residual1-residual4~IW(0,13);!degrees of freedom =13!
residual5-residual7~IW(0,13);!degrees of freedom =13!
residual8-residual28~IW(0,13);!degrees of freedom =13!
Output:
```

```
    STAND(STDYX) sampstat stand tech4 stdyx tech8;
Plot: type=plot2;
```
